# Activatable red/near-infrared aqueous organic phosphorescence probes for improved time-resolved bioimaging

**DOI:** 10.1093/nsr/nwae383

**Published:** 2024-10-29

**Authors:** Yang Li, Zhiqin Wu, Zizhao Huang, Chenjia Yin, He Tian, Xiang Ma

**Affiliations:** Key Laboratory for Advanced Materials and Joint International Research Laboratory of Precision Chemistry and Molecular Engineering, Frontiers Science Center for Materiobiology and Dynamic Chemistry, School of Chemistry and Molecular Engineering, East China University of Science and Technology, Shanghai 200237, China; Key Laboratory for Advanced Materials and Joint International Research Laboratory of Precision Chemistry and Molecular Engineering, Frontiers Science Center for Materiobiology and Dynamic Chemistry, School of Chemistry and Molecular Engineering, East China University of Science and Technology, Shanghai 200237, China; Key Laboratory for Advanced Materials and Joint International Research Laboratory of Precision Chemistry and Molecular Engineering, Frontiers Science Center for Materiobiology and Dynamic Chemistry, School of Chemistry and Molecular Engineering, East China University of Science and Technology, Shanghai 200237, China; Key Laboratory for Advanced Materials and Joint International Research Laboratory of Precision Chemistry and Molecular Engineering, Frontiers Science Center for Materiobiology and Dynamic Chemistry, School of Chemistry and Molecular Engineering, East China University of Science and Technology, Shanghai 200237, China; Key Laboratory for Advanced Materials and Joint International Research Laboratory of Precision Chemistry and Molecular Engineering, Frontiers Science Center for Materiobiology and Dynamic Chemistry, School of Chemistry and Molecular Engineering, East China University of Science and Technology, Shanghai 200237, China; Key Laboratory for Advanced Materials and Joint International Research Laboratory of Precision Chemistry and Molecular Engineering, Frontiers Science Center for Materiobiology and Dynamic Chemistry, School of Chemistry and Molecular Engineering, East China University of Science and Technology, Shanghai 200237, China

**Keywords:** organic room-temperature phosphorescence, activatable molecular probe, host–guest, time-resolved imaging, *in vivo* biosensing

## Abstract

Organic red/near-infrared (NIR) room-temperature phosphorescence (RTP) holds significant potential for autofluorescence-free bioimaging and biosensing due to its prolonged persistent luminescence and exceptional penetrability. However, achieving activatable red/NIR organic RTP probes with tunable emission in aqueous solution remains a formidable challenge. Here we report on aqueous organic RTP probes with red/NIR phosphorescence intensity and lifetime amplification. These probes consist of supramolecular assemblies comprising macrocyclic cucurbit[8]uril and amine-containing alkyl-bridged pyridiniums, exhibiting viscosity-activatable phosphorescence with enhanced quantum yield (≤20%) and lifetime. Notably, by utilizing this activatable organic RTP probe, we successfully achieve two-photon imaging of lysosomal viscosity and millisecond-scale time-resolved cell imaging. Moreover, intravital phosphorescence imaging by using an RTP probe enables the monitoring of viscosity variations in inflammatory mice, demonstrating a significantly improved signal-to-background ratio compared with fluorescence imaging. This activatable red/NIR supramolecular platform facilitates versatile high-resolution phosphorescence imaging for *in vivo* tracking of specific biomarkers and physiological events.

## Teaser

The construction of organic activatable RTP probes with amplified phosphorescence emission intensity and lifetime was achieved, enabling unprecedented visible-light excited phosphorescence imaging at both cellular and in vivo levels.

## INTRODUCTION

Optical imaging plays an essential role in fundamental biological and biomedical research, as well as clinical applications [1]. Until now, photoluminescence (PL) has remained the predominant modality that is employed in optical bioimaging, including fluorescence and phosphorescence [2]. In general, phosphorescence emission exhibits longer lifetimes (microseconds to seconds) compared with fluorescence (nanoseconds to microseconds) due to the occurrence of spin-forbidden transitions from excited triplet states back to ground states [3–6]. Consequently, phosphorescence can manifest discernible afterglow even upon cessation of the light source [7–9]. Additionally, organic room-temperature phosphorescent (RTP) probes possess advantageous attributes such as minimal toxicity and excellent biocompatibility [10]. The advancement of organic RTP probes would greatly benefit autofluorescence-free bioimaging and theranostics [11–13]. To date, diverse strategies have been employed for the construction of organic RTP probes, such as supramolecular assemblies and the formation of phosphorescent nanoparticles [14–16]. However, the majority of current organic RTP probes suffer from limited cell and tissue penetration due to their ultraviolet (UV) light excitation and short phosphorescent emission wavelengths (<600 nm), thereby hindering the accurate acquisition of physiological and pathological information [17,18]. Recently, red/near-infrared (NIR) organic phosphorescence has facilitated *in vivo* bioimaging by enabling augmented tissue penetration [19–21]. Nonetheless, organic RTP probes commonly lack specific interactions with the target of interest, resulting in a restricted imaging signal-to-background ratio (SBR) and detection sensitivity [22–24]. Thus, the precise visualization of biological events or anatomical features of interest by using organic red/NIR phosphorescence probes remains challenging.

To overcome this limitation, the utilization of activatable red/NIR molecular probes that exhibit phosphorescence emission has become indispensable, as they enable signal amplification for specific biomarkers and thereby enhance the sensitivity of organic phosphorescence detection while simultaneously improving spatio-temporal resolution in bioimaging [25–28]. Host–guest complexes are ideal candidate probes for this purpose due to their available aqueous RTP and tunable absorption/emission wavelengths [29–34]. However, the development of activatable red/NIR organic RTP probes mainly lacks effective material design strategies, thereby significantly impeding high-contrast phosphorescence imaging and sensing applications.

**Figure 1.**
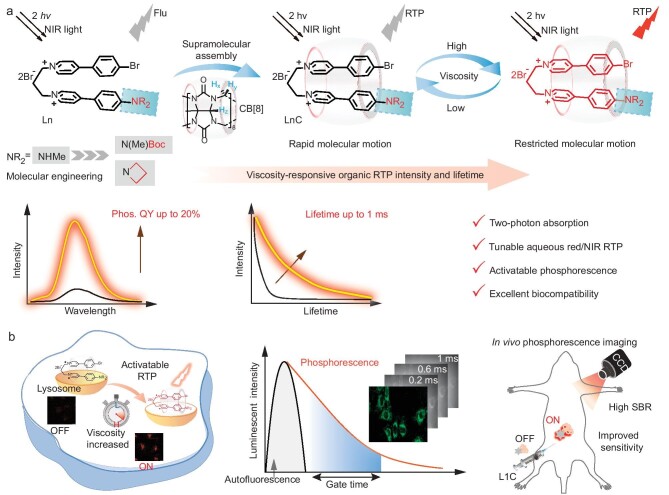
Supramolecular engineering for activatable organic RTP probes with improved emission in aqueous solution. (a) Work principle of viscosity-activatable red/NIR organic RTP probe via supramolecular assembly between amine-containing alkyl-bridged pyridiniums molecular scaffold and CB[8]. (b) Activatable organic RTP probes for versatile phosphorescence imaging and sensing applications. flu, fluorescence; phos., phosphorescence; QY, quantum yield.

Here, we present an activatable red/NIR organic RTP probe that was achieved through the combination strategy of assembly-induced emission and adjustable twisted intramolecular charge transfer (TICT). These RTP probes were synthesized through the tailored host–guest assembly of macrocyclic cucurbit[8]uril (CB[8]) and amine-containing alkyl-bridged pyridiniums (Fig. 1a). We initially investigated the impact of molecular modification and structural parameters on the phosphorescence performance of these supramolecular assemblies, while also proposing a plausible mechanism for a viscosity-activatable RTP probe. By engineering the guest molecule through the amino site, we demonstrated tunable red/NIR phosphorescence with an improved quantum yield (QY) and extended lifetime in highly viscous environments. Finally, we evaluated the optical performance of activatable organic RTP probes in various bioimaging and biosensing applications (Fig. 1b), encompassing lysosome-targeted two-photon imaging, monitoring cellular lysosomal viscosity, time-resolved cell imaging and intravital phosphorescence imaging for detecting viscosity changes in inflammatory mouse model. The access to activatable red/NIR phosphorescence probes provides a rational approach for the design of aqueous-phase organic RTP probes with bright triplet excitons, potentially unlocking previously unexplored optical imaging and sensing applications that are facilitated by these probes.

## RESULTS

### Molecular design and photophysical properties

In order to enhance the applicability of aqueous RTP probes while optimizing their optical properties for bioimaging, our group has focused on the development of high-performance phosphorescent materials by using assembly-induced emission strategies, such as leveraging host–guest systems [35–38]. Our design strategy for the activatable RTP probe involves combining a viscosity-responsive dye that exhibits the TICT effect within a host–guest system that is capable of generating aqueous phosphorescence (Scheme S1). We selected alkyl-bridged pyridinium as the preferred guest molecule due to its tunable TICT and its ability to exhibit aqueous phosphorescence upon assembly with the host CB[8] [39,40]. To explore viscosity-activated probes with improved phosphorescence performance, we introduced a *tert*-butoxycarbonyl (Boc) group to replace the amino group in alkyl-bridged pyridinium **L3**, resulting in the synthesis of guest molecule **L1** (Fig. 1). Additionally, we utilized the structural rigidity of azetidine to moderately attenuate the TICT that was exhibited by the alkylamino of **L3**.

The guest molecules **Ln** (*n* = 1–3) were synthesized via Suzuki coupling and nucleophilic substitution reactions by using a commercially available pyridine borate compound (Scheme S2). The guest molecules in this series were successfully identified and characterized by using ^1^H and ^13^C nuclear magnetic resonance (NMR) spectroscopy, as well as high-resolution mass spectrometry (Figs S1–S13). The UV–vis absorption spectra of the guest molecules **Ln** (*n* = 1–3) revealed that their respective maximum absorption wavelengths were 310, 425 and 420 nm (Fig. S14). The steady-state spectra of **Ln** (*n* = 1–3) showed that their fluorescent emission peaks were situated within the range of 400–600 nm (Fig. S15). Following the initial measurement of the luminescent property of the **Ln** guests, the aqueous solution of the guests was added with host CB[8], which is a water-soluble macrocycle compound that is capable of encapsulating the pyridinium unit and inducing phosphorescence.

**Figure 2.**
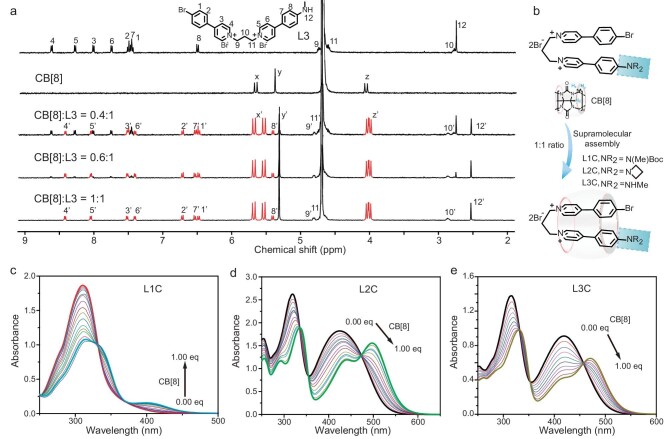
Characterization of supramolecular assembly behavior. (a) ^1^HNMR spectral changes of **L3** after adding 0, 0.4, 0.6 and 1.0 equivalent CB[8] (0.1 mM, D_2_O). (b) Schematic diagram of a 1:1 supramolecular assembly complex formed by **Ln** (*n* = 1–3) and CB[8]. (c–e) UV–vis absorption spectra of **Ln** (*n* = 1–3) and CB[8] at concentrations ranging from 0 to 1 × 10^−4^ M in water.

The assembly behavior of **Ln** and CB[8] was investigated through ^1^H NMR titration spectroscopy (Fig. 2a and Figs S16 and S17). The insights into the binding behaviors were obtained through ^1^H NMR spectroscopy, revealing that the chemical shifts of the phenyl protons and pyridiniums of **Ln** experienced upfield shifts while the alkyl protons exhibited downfield shifts. These observations suggested that the aromatic moieties were encapsulated within the CB[8] whereas the linked alkyl chain resides outside. Furthermore, upon the addition of CB[8] to **L1**, the peak at ∼310 nm showed a decrease in intensity, concomitantly with the emergence of a novel peak at ∼405 nm. Additionally, there was an observable transition in the solution color from colorless to a faint yellow (Fig. 2c and Fig. S18). The supramolecular assemblies between **L2** and **L3** with CB[8] also exhibited significant color changes. (Fig. 2d and e). These results suggested the formation of a highly efficient charge-transfer assembly of **Ln** upon binding with CB[8]. Additionally, the binding behavior between **Ln** (*n* = 1–3) and CB[8] was assessed by using Job's plots. The plots indicated a 1:1 ratio for the binding of **Ln** (*n* = 1–3) with CB[8] macrocycle in water (Fig. S19). The formation of a 1:1 supramolecular assembly was further supported by using high-resolution electrospray ionization-time of flight mass spectrometry (ESI-TOF MS) analysis (Figs S20–S22) and the observed molecular weight for the complexes closely corresponded to the calculated 1:1 ratio. In conjunction with the outcomes of the high-resolution MS, UV–vis spectra titrations and ^1^HNMR spectroscopy, we substantiated that **Ln** (*n* = 1–3) effectively adopts a folded conformation within the CB[8] cavity to establish a 1:1 host–guest assembly mode (Fig. 2b).

**Figure 3.**
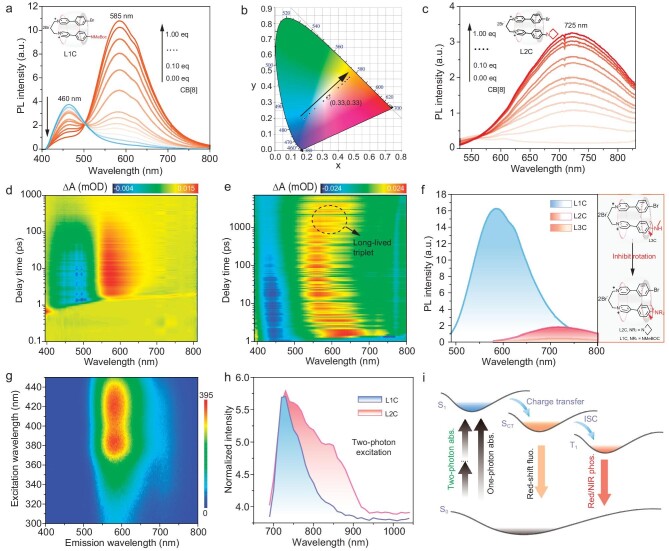
Photophysical properties of assemblies **LnC**. (a) PL spectra of **L1** (0.1 mM) and CB[8] (excited by 360 nm) aqueous solutions at concentrations ranging from 0 to 0.09 mM. (b) Chromaticity coordinate (CIE) of **L1C** with varying CB[8] ratios in aqueous solution in accordance with (a). (c) PL spectra of aqueous solutions of **L2** (0.1 mM) and CB[8] (excited by 500 nm) at concentrations ranging from 0 to 0.09 M. (d) and (e) Femtosecond broadband-TA spectra of **L1C** and **L2C** in water (340 nm excitation). (f) Comparison of PL spectra of **L1C**–**L3C** at the same concentration (0.1 mM); structural regulation of the N-terminal of **LnC** supramolecular platform. (g) Phosphorescence-excitation emission mapping of **L1C** (0.1 mM). (h) Two-photon excitation spectra of **L1C** (0.1 mM) and **L2C** (0.1 mM) in water. (i) A potential schematic of the NIR excitation and emission mechanism in **L1C-L3C** systems. abs., absorption; ISC, intersystem crossing.

To explore the changes in the luminescence during the assembly process of guest molecules **Ln** (*n* = 1–3) and CB[8], we conducted measurements on their PL spectra. Upon the addition of CB[8], novel emission peaks (550–800 nm, λ_L1_ ≈ 585 nm, λ_L2_ ≈ 725 nm and λ_L3_ ≈ 720 nm) were observed in the PL spectra (Fig. 3a and c, and Fig. S23). The Commission International del'Eclairage coordinates were conducted to illustrate the color change of the PL emission of the complex **L1C** upon the addition of CB[8]. Figure 3b clearly demonstrates a transition from blue to orange–red, passing through a white-light region. The time-resolved PL spectra provided evidence of the presence of phosphorescent emission. The lifetimes of red/NIR emissions were significantly improved upon deoxygenation through argon bubbling, in accordance with the phenomenon that oxygen quenches triplet-state electrons (Fig. S24). Moreover, we compared the emission intensities of **L1C, L2C** and **L3C**, and observed a significantly lower emission intensity for **L3C** in comparison with both **L1C** and **L2C**. This result suggested that the rigidity of the azetidinyl and the electron-withdrawing nature of the Boc group may impede the rotation of the amino group, thereby reducing the non-radiative decay processes and resulting in an enhanced phosphorescence emission intensity (Fig. 3f). The amine group in this supramolecular platform can serve as a site for phosphorescence regulation.

Femtosecond transient absorption (TA) of the supramolecular assemblies **LnC** in aqueous solution were explored and are presented in Fig. 3d and e, and Fig. S25 to further elucidate the optical processes. **L1C** exhibited an excited-state absorption (ESA) signal, which was attributed to singlet absorption, within the wavelength range of 600–700 nm immediately following excitation. This signal subsequently transformed into a long-life broad ESA band that spanned from 500 to 650 nm, concomitantly with a concurrent reduction in the stimulated emission (SE) signal that it centered around. The emerging ESA signal was attributed to the triplet ESA, as evidenced by its continuous increase throughout the entire detection window. The absence of a ground-state bleach signal for the assemblies can be attributed to the absorption being beyond our test range. A strong SE signal at ∼450 nm persisted throughout the entire detection time window of 1000 ps for **L2C**, with a noticeable increase in intensity observed (Fig. S25). Following excitation, only ESA within the range of 550–700 nm, which was attributed to singlet state absorption, was observed.

Excitation-phosphorescence emission mapping of the **L1C** assembly revealed that its optimal excitation wavelength falls within the visible-light range (Fig. 3g). The PL spectra of **L2C** exhibited a wavelength-dependent shift in emission, accompanied by corresponding changes in the luminescence color upon excitation with different wavelengths (Fig. S26). Furthermore, the NIR two-photon absorption (TPA) performance of probes **L1C** and **L2C** was subsequently evaluated, considering their potential for enhanced tissue penetration and reduced photodamage in biological applications. Figure 3h demonstrates that **L1C** and **L2C** exhibited NIR TPA in water (using Rhodamine B as a standard and ethanol as the optimized solvent). Consequently, these assemblies possess both two-photon NIR excitation and red/NIR phosphorescence emission (Fig. 3i).

### Viscosity-responsive performance of the RTP probe LnC

**Figure 4.**
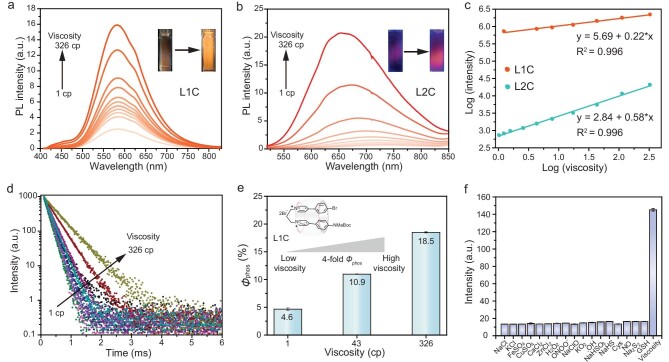
Viscosity response performance of probe **LnC**. (a) PL spectra of **L1C** (50 μM) in water/glycerol mixtures with different viscosities in the emission window of 400–800 nm (excitation wavelength: 400 nm). Inset: photographs of **L1C** (50 μM) in water and water/glycerol mixtures under 395 nm of light irradiation under ambient conditions. (b) PL spectra of **L2C** (50 μM) in water/glycerol mixtures with different viscosities in the emission window of 520–850 nm (excitation wavelength: 500 nm). Inset: photographs of **L2C** (50 μM) in water and water/glycerol mixtures under 450 nm of light irradiation under ambient conditions. (c) Linear relationship between log phosphorescence and log η of **L1C** and **L2C**. (d) Phosphorescence lifetime spectra of **L1C** (50 μM) in the water/glycerol solutions, viscosity ranging from 3 to 326 cP. (e) The phosphorescence QY of **L1C** (50 μM) at different viscosities. (f) Phosphorescence response of **L1C** (50 μM) to various metal ions, ROS and RNS (5 mM NaCl, 5 mM KCl, 200 μM FeSO_4_, 200 μM CuSO_4_, 200 μM CaCl_2_, 200 μM ZnCl_2_, 1 mM H_2_O_2_, 10 μM ONOO^−^, 10 μM NaClO, 10 μM KO_2_, 10 μM •OH, 10 μM NO, 10 μM NaHSO_3_, 10 μM NaHS, 10 μM Na_2_S_2_, 100 μM Cys, 100 μM GSH, 95% glycerol).

The absorption and PL spectra of supramolecular assemblies **LnC** in water/glycerol solutions with varying viscosities were subsequently investigated. The absorption intensity of the probe **LnC** (*n* = 1–3) indicated a slight increase with increasing viscosity (Fig. S27). The results, which are depicted in Fig. 4a and b and Fig. S28, show heightened emission intensities of **LnC** (*n* = 1–3) within high-viscosity solutions, whereas low-emission or non-radiative behavior is observed at relatively lower viscosities. Significantly, upon one-photon excitation at 400 nm, **L1C** exhibited a remarkable 8-fold enhancement in phosphorescence emission within the wavelength range of 400–800 nm as the viscosity increased from 3.0 to 326.0 cp. The phosphorescence intensity of **L1C** at 585 nm (log_phos._) showed a strong linear correlation with the viscosity of the medium (log*η*, 3.0–326.0 cp), demonstrating its potential as a reliable indicator. Furthermore, **L2C** demonstrated a remarkable 26-fold enhancement in phosphorescence and also displayed a linear relationship (Fig. 4c). The phosphorescence lifetime of **L1C** exhibited a similar viscosity-dependent trend (Fig. 4d). Additionally, a linear correlation between log *τ* and log *η* was observed, aligning with the Förster–Hoffmann equation across a range of viscosities from 3.0 to 326.0 cp (Fig. S29). The phosphorescence lifetime of **L1C** was significantly prolonged with the increase in viscosity in the mixed solution. The phosphorescence QY (*Φ*_p_) of **L1C** in water was determined to be 4.6% (Fig. 4e and Fig. S30). Interestingly, when dissolved in a highly viscous solution (95% glycerol), the phosphorescence QY of **L1C** increased significantly to 18.5%, exhibiting a 4-fold enhancement compared with that observed in water. The QY of **L2C** in the aqueous phase was measured at 0.3% while a higher viscosity resulted in a QY of 1.2% for **L2C** (Fig. S31). The viscosity environment may impede the molecular motion of **LnC** series supramolecular assemblies, thereby reducing non-radiative decay and consequently enhancing phosphorescence emission (Fig. S32). The above results validate the exceptional sensitivity of **LnC** towards viscosity and its robust red/NIR phosphorescence.

The temperature-responsive phosphorescence emission of **L1C** in high-viscosity solvents (405 cp) was investigated. The temperature-dependent phosphorescence spectra of **L1C** were investigated in the range of 293–353 K, with the dominant emission observed at 293 K being attributed to RTP. Notably, a strong linear correlation between the phosphorescence intensity of **L1C** and temperature (273–353 K) was observed in 99% water/glycerol mixtures (Fig. S33). The phosphorescence lifetime of **L1C** demonstrates an exponential decay relationship with temperature, as depicted in Fig. S33. Notably, at 0°C, the lifetime of **L1C** exceeds 1 ms. This finding suggests that **L1C** with high viscosity demonstrates temperature sensitivity while maintaining its excellent phosphorescent properties even at elevated temperatures.

To investigate the specificity of **L1C** and **L2C** towards viscosity, we evaluated their selectivity by monitoring reactions with various reactive oxygen species (ROS), metal ions and reactive nitrogen species (RNS) under simulated physiological conditions. Encouragingly, only an increase in high viscosity resulted in a significant enhancement of the red phosphorescence signal without any interference from other highly abundant RNS, ROS, metal ions, proteins and nucleic acids (Fig. 4f and Figs S34 and S35). We also observed that the phosphorescence intensity of **L1C** cannot be enhanced by altering the environmental polarity (Fig. S36). This observation unequivocally demonstrates the good specificity of **L1C** and **L2C** towards viscosity. Moreover, the phosphorescence intensity of **L1C** remained consistently stable across various pH values in phosphate buffered saline (PBS). Additionally, upon the addition of glycerol, **L1C** and **L2C** revealed a significant enhancement of red phosphorescence over a wide pH range (pH 5.0–9.0) without any notable variations, thereby indicating their excellent pH stability (Figs S37 and S38).

### Cellular phosphorescence imaging

**Figure 5.**
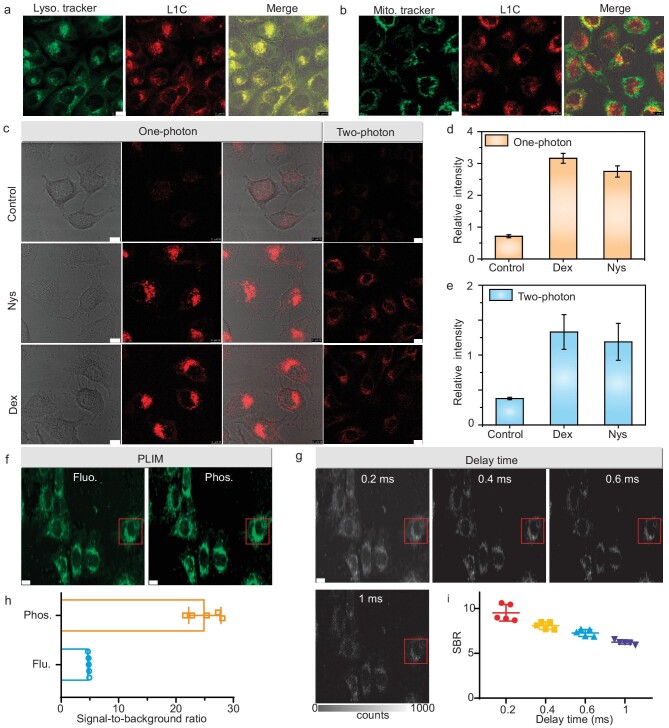
Cellular phosphorescence imaging and viscosity detection by **L1C**. (a) and (b) Colocalization images of HeLa cells co-incubated with **L1C** for the red channel (50 μM, collected from 580 to 650 nm, excitation wavelength = 405 nm) and the corresponding organelle targeting dyes for the green channel (LysoTracker Green, 70 nM, collected from 490 to 530 nm, excitation wavelength = 485 nm; MitoTracker Green, 50 nM, collected from 490 to 530 nm, excitation wavelength = 485 nm). Scale bar: 10 μm. (c) The HeLa cells were incubated with **L1C** (50 μM) only and the cells were pretreated with 20 μM of dexamethasone (Dex) for 20 min and then incubated with **L1C** (50 μM) for another 40 min; the cells were pretreated with 20 μM of nystatin (Nys) for 20 min and then incubated with **L1C** (50 μM) for another 40 min. One-photon imaging: *λ*_ex_ = 405 nm, *λ*_em_ = 580–700 nm, scale bar: 10 μm. Two-photon imaging: *λ*_ex_ = 720 nm, *λ*_em_ = 580–700 nm, scale bar: 25 μm. (d) and (e) Phosphorescence intensities of images in (c), which were obtained by using ImageJ. Fluorescent and long-lived phosphorescent images (f) and time-gated luminescent images with various delay times (g) of HeLa cells treated with **L1C** for 4 h. Scale bar: 20 μm. (h) and (i) Phosphorescence and fluorescence intensity analysis of images in (f) and (g) with ImageJ software.

Motivated by the photophysical properties of RTP probe **L1C**, we subsequently investigated its potential for cellular phosphorescence imaging and viscosity detection. Considering the substantial demand for a phosphorescence imaging probe with exceptional biocompatibility, we further ascertained that **L1C** demonstrated negligible cytotoxicity (Fig. S39). Moreover, the intracellular photostability of **L1C** was assessed by subjecting it to continuous irradiation by using a 405-nm laser in a confocal high-resolution microscope for a duration of 480 s. Importantly, no discernible fluctuations in the red phosphorescence intensity of **L1C** were observed upon exposure to the 405-nm laser for 420 s. These findings indicate that **L1C** demonstrates remarkable intracellular photostability and resistance against photobleaching (Fig. S40). To elucidate the subcellular localization of **L1C** in phosphorescence imaging, we co-stained HeLa cells with **L1C** alongside commercially available organelle dyes such as Lysosome Tracker Green and Mitochondria Tracker Green. As depicted in Fig. 5a and b, **L1C** showed accumulation in lysosomes of HeLa cells, displaying red dot phosphorescence that showed excellent colocalization (colocalization coefficient = 0.85) with lysosomal green fluorescence. However, the colocalization coefficient with mitochondria was only 0.37. The high charge density and strong electrostatic repulsive interactions of **L1C** assemblies likely altered their lipophilicity and reduced electrophoretic force, thereby favoring lysosomal localization [10]. Overall, our findings demonstrated specific targeting of HeLa cell lysosomes by **L1C** while maintaining a stable phosphorescent intensity.

The local viscosity of lysosomes has been reported to increase following treatment with ionophores such as dexamethasone and nystatin [41–43]. Therefore, we evaluated the potential of **L1C** for detecting dynamic changes in lysosomal viscosity under dexamethasone and nystatin stimulation. HeLa cells were incubated with 20 μM of dexamethasone and nystatin, followed by staining with organic RTP probe **L1C**. As depicted in Fig. 5c and d, the one-photon and two-photon excited red phosphorescence within lysosomes in HeLa cells exhibited a significant enhancement upon stimulation with dexamethasone and nystatin. Compared with the control group with only **L1C** assembly added, HeLa cells pretreated with dexamethasone and nystatin displayed a more than three times increase in phosphorescence intensity (Fig. 5e and f). These findings demonstrated that **L1C** enabled real-time imaging of dynamic changes in lysosomal viscosity under the influence of dexamethasone and nystatin.

To enable precise microsecond and millisecond time-gated imaging in a cellular lever by using probe **L1C**, we conducted phosphorescence lifetime imaging microscopy (PLIM). Figure 5f demonstrates the simultaneous detection of fluorescent and phosphorescent signals of **L1C** in HeLa cells. The SBR for the phosphorescent image (SBR = 25.0) was more than five times higher than that of the fluorescent image (SBR = 4.8) (Fig. 5h). Additionally, the long-life phosphorescent signal with a lifetime of >1 ms was clearly detectable (Fig. 5g), indicating that **L1C** serves as an effective probe for obviating short-life autofluorescence from the background. By increasing the delay time beyond 50 ns (0.2, 0.4, 0.6 and 1 ms), the short-life fluorescent signal is comprehensively filtered out while maintaining stable detection of the long-life phosphorescent group signal with higher SBR values (SBR = 10.0, 8.1, 7.3 and 6.3) (Fig. 5i). Therefore, this RTP probe **L1C** will be promising in real-time phosphorescent imaging of the complicated biological environment.

### *In vivo* phosphorescence bioimaging and biosensing

**Figure 6.**
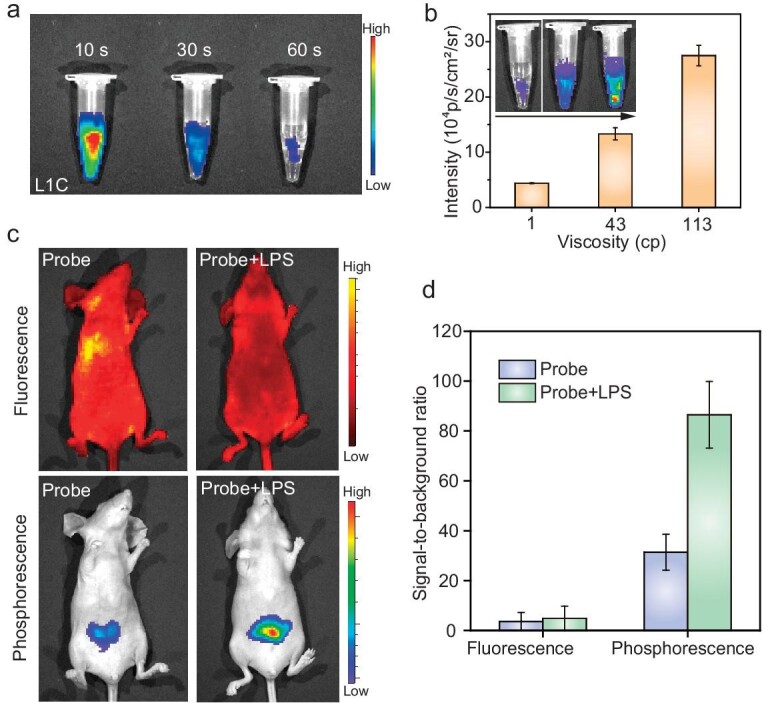
*In vivo* phosphorescence biosensing application. (a) Afterglow images after 450 nm of visible-light irradiation (120 mW/cm^2^) of probe **L1C** (500 μM) for 10 s. (b) Phosphorescence signals of probe **L1C** (200 μM) in different solution viscosities. (c) Phosphorescence and fluorescence imaging after the intraperitoneal injection of probe **L1C** (300 μM) into LPS-induced inflammatory mouse model and normal control group. (d) SBR for phosphorescence and fluorescence imaging of LPS-induced inflammatory mouse model and normal control group. All error bars were based on standard deviation (*n* = 3).

The activatable phosphorescence intensity and lifetime of probe **L1C** prompted us to investigate its potential for *in vivo* biosensing. Intravital afterglow bioimaging was performed by using an *in vivo* imaging system (IVIS) in bioluminescence mode under 450 nm of visible-light irradiation. For comparison, fluorescence signals that were derived from probe **L1C** were also simultaneously evaluated. As exhibited in Fig. 6a, a detectable phosphorescence signal from probe **L1C** persisted for 60 s after the cessation of visible-light excitation. Furthermore, the phosphorescence signal of **L1C** significantly increased as the viscosity of the solution increased (Fig. 6b), aligning with the luminescence spectrum results. Additionally, **L1C** exhibited remarkable resistance to photobleaching, indicating minimal intensity changes after eight cycles of 450 nm of visible-light irradiation (Fig. S41). The *in vitro* phosphorescence imaging results indicated the promising potential of probe **L1C** for afterglow bioimaging.

The afterglow imaging of subcutaneously injected probe **L1C** in nude mice (Fig. S42) demonstrated the detection of phosphorescent signals within 10 s after the laser removal. Subsequently, a viscosity-altering mouse model was constructed by administering lipopolysaccharide (LPS) to induce inflammation and increase viscosity [44,45] (Fig. 6c). Compared with the control group that were injected with probe only, LPS-treated mice exhibited a 2.7-fold higher phosphorescence intensity (Fig. 6d), indicating significant variations in viscosity under LPS-induced inflammatory conditions. In contrast, the fluorescence signals of probe **L1C** were indistinguishable from tissue autofluorescence, rendering accurate pathological information unattainable. This study validates the potential of activatable RTP probe **L1C**, possessing both long-lifetime and long-wavelength properties, as a robust *in vivo* biosensing tool by effectively mitigating autofluorescence interference and optimizing response performance.

## CONCLUSION

In summary, we have successfully synthesized activatable red/NIR organic RTP probes by employing the supramolecular assembly between macrocyclic CB[8] and guest molecules **LnC** (*n* = 1–3). The secondary amino group within the **LnC** molecular framework can serve as an aqueous RTP regulatory site, while modification with Boc and azetidinyl groups enhances phosphorescence emission. The red/NIR phosphorescence intensity and lifetime of **LnC** assemblies showed a significant enhancement with an increase in the solution viscosity. Moreover, probe **L1C** exhibited exceptional stability, biocompatibility and specificity towards viscosity response. Notably, probe **L1C** demonstrated specific targeting of lysosomes, millisecond-scale time-resolved cellular phosphorescence imaging and two-photon imaging of intracellular viscosity. Importantly, *in vivo* phosphorescence imaging revealed that probe **L1C** enabled real-time monitoring of viscosity fluctuations in inflammatory mice with a high SBR. Therefore, the findings that are presented in this study are expected to make a significant contribution to the advancement of high-contrast phosphorescence imaging for the real-time visualization of vital physiological processes in cells, as well as *in vivo* biosensing.

## MATERIALS AND METHODS

All reagents and solvents were used as received from commercial sources without further purification, unless otherwise specified. Femtosecond transient absorption spectra were acquired by using the Time-Tech Spectra TA100 instrument. Two-photon confocal imaging was performed on a Leica TCS-SP8 fluorescence microscope that was equipped with an oil immersion objective. The PLIM set-up was integrated with an Olympus IX81 laser scanning confocal microscope. *In vivo* phosphorescence imaging was conducted by using the IVIS Lumina II imaging system. The animal procedures were conducted in strict accordance with the Guidelines for Care and Use of Laboratory Animals of East China University of Science and Technology.

### The Förster–Hoffmann equation

The Förster–Hoffmann equation was utilized to correlate the relationship between the phosphorescence emission intensity and lifetime of **LnC** and the solvent viscosity: log*P* = *k*log*η* + *C*, in which *η* is the medium viscosity, *P* is the phosphorescence emission intensity, *C* is the concentration and temperature constant, and *k* is the dye-dependent constant. log*τ* = *k*log*η* + *C*, in which *η* is the medium viscosity and *τ* is the lifetime.

### Colocalization cell imaging

The HeLa cells were seeded onto a confocal Petri dish and cultivated for 24 h at 37°C in a 5% CO_2_ environment. Subsequently, the cells were incubated with the **L1C** solution (5 × 10^−5^ M) for 6 h. Following removal of the medium, the cells were washed three times with PBS solution. Next, MitoTracker Green or LysoTracker Green was co-cultured with the cells at 37°C for 20 min to stain the mitochondria or lysosomes, respectively. After being repeatedly washed at least three times with PBS, the cells were subjected to observation by using a confocal microscope.

### Phosphorescence imaging of cellular viscosity

The HeLa cells were pretreated with dexamethasone and nystatin (20 μM) for 20 min, respectively. Subsequently, all the groups of cells were stained with **L1C** (50 μM) for 40 min and then washed three times with 1.0 mL of PBS. Finally, confocal imaging was performed on the cells by using a Leica SP8 high-resolution fluorescence microscope and Leica Application Suite X software.

### PLIM cell imaging

The HeLa cells were cultured with **L1C** (50 μM) for 6 h. Following incubation, the cells were washed three times with PBS. Subsequently, phosphorescence cell images were acquired by using PLIM through integration with an Olympus IX81 laser scanning confocal microscope. The confocal microscope system detected the phosphorescence signals and data analysis was performed by using professional software that was provided by PicoQuant Company. Double exponential tailfit was applied to fit the PLIM data that were obtained from the regions of interest, sized at 256 × 256 pixels, with a binning factor of 1 in SymPhoTime 64 pro software.

## Supplementary Material

nwae383_Supplemental_File

## Data Availability

The corresponding author can provide all relevant data supporting the findings of this study upon reasonable request.
