# Supplementary material for: Activatable red/near-infrared aqueous organic phosphorescence probes for improved time-resolved bioimaging
Source: Natl Sci Rev. 2024 Oct 29;12(2):nwae383. doi: 10.1093/nsr/nwae383 (PMC11737404; doi:10.1093/nsr/nwae383)
Supplement: nwae383_Supplemental_File [file nwae383_supplemental_file.pdf]

Supplementary information

Activatable red/near-infrared aqueous organic phosphorescence probes for improved time-resolved bioimaging

Yang Li, Zhiqin Wu, Zizhao Huang, Chenjia Yin, He Tian, Xiang Ma*

Key Laboratory for Advanced Materials and Joint International Research Laboratory of Precision Chemistry and Molecular Engineering, Frontiers Science Center for Materiobiology and Dynamic Chemistry, School of Chemistry and Molecular Engineering, East China University of Science and Technology, Shanghai 200237

Contents

Experimental section S2

Figures S1–S13. Synthesis and compound characterization S4

Figures S14–S21. Binding behaviors of **LnC** (n=1-3) S15

Figures S22–S25. Optical properties of **LnC** (n=1-3) S18

Figures S26–S30. Viscosity response of **LnC** (n=1-3) S20

Figures S31–S35. Bioimaging applications S26

References S27

Experimental section

Instrumentation and methods. All reagents and solvents were commercially available and used without further purification unless otherwise noted. ^{1}H NMR and ^{13}C NMR spectra were measured on a Bruker AV-400 spectrometer. The electronic spray ionization (ESI) high-resolution mass spectra were tested on a Waters LCT Premier XE spectrometer. The UV-Vis absorption spectra were obtained on a Shimadzu UV-2600 spectrophotometer. Fluorescence, phosphorescence, and lifetime of delayed emission spectra were recorded on an Agilent Cary Eclipse spectrophotometer. Phosphorescence mode; Delay time = 0.1 ms. Photoluminescence spectra were record on Shimadzu RF-6000 spectrometer. Fluorescence lifetimes were measured on Edinburgh Instruments Fluorescence Spectrometer (FLS1000). Absolute PL quantum yields were determined with a spectrometer C11347-11 (Hamamatsu, Japan). Femtosecond transient absorption (TA) spectra were obtained on Time-Tech Spectra TA100. Leica TCS-SP8 fluorescence microscope equipped with an oil immersion objective (63×) was used for two-photon confocal imaging. The phosphorescence lifetime imaging microscope (PLIM) image setup was integrated with an Olympus IX81 laser scanning confocal microscope. IVIS Lumina II imaging system was used for in vivo phosphorescence imaging.

Determination of binding stoichiometry. The total concentration of **Ln** (n=1-3) and CB[8] in Job plot experiments was maintained at 0.02 mM. UV absorption spectra were recorded by varying the **Ln** molar ratio from 0.1 to 0.9, while simultaneously adjusting the CB[8] molar ratio from 0.9 to 0.1. Control experiments involved measuring the absorption of **Ln** solutions with the same concentration but without CB[8]. The absorbance subtraction at 310 nm was utilized for calculating the binding ratio between **Ln** and CB[8].

Measurement of viscosity. The solutions of **LnC** (final concentration 50 μM) with varying viscosities were prepared by adding the stock solution (1 mM) of **LnC** to a mixture (2 mL) of water-glycerol with different volume ratios. The solutions were

continuously shaken for 1 hour, and after allowing them to stand for 30 minutes to remove any air bubbles, their emission spectra were measured. The viscosity values of the water-glycerol mixtures at different proportions and temperatures (25 ± 0.1) ℃ are presented in Table S1.

Cell culture and cytotoxicity experiments. The HeLa cells were cultured in Gulbecco's modified Eagle medium (DMEM) supplemented with 10% fetal bovine serum (FBS) and 1% penicillin/streptomycin. The incubation of HeLa cells was performed using DMEM medium containing 10% FBS and 1% penicillin/streptomycin, under a humidified atmosphere with 5% CO_2 at 37℃. Subsequently, the cells were treated with different concentrations of guest and host-guest complexes **L1C** for a duration of 24 hours. The relative cellular viability was assessed using the MTT assay following the provided instructions.

In vitro phosphorescent imaging. The phosphorescent intensities of probe **L1C** were recorded using IVIS® Lumina II imaging system at t= 10 s after the solutions were irradiated by 450 nm handheld blue lamp (10 W) for 10 s. The IVIS system was set in bioluminescence mode with an open filter setting (exposure time: 20 s).

Phosphorescence imaging of viscosity *in vivo.* The animal procedures were conducted in strict accordance with the Guidelines for Care and Use of Laboratory Animals of East China University of Science and Technology. Female nude mice at six weeks old were obtained from Shanghai Yishang Biotechnology Co., Ltd. Each group consisted of three mice. To validate the suitability of RTP probe **L1C** for *in vivo* viscosity monitoring, the mice were divided into two groups and injected with lipopolysaccharide (LPS) in their abdomen to induce changes in viscosity. In the first group, **L1C** (0.3 mM) was administered intraperitoneally as a negative control. The second group received an intraperitoneal injection of LPS (100 μL, 1 mg/mL) followed by intraperitoneal injection of **L1C** (0.3 mM) after 24 hours. Fluorescent images were acquired with excitation at 460 nm and emission at 620 nm (exposure

time: 0.1 s). Phosphorescence images were obtained after irradiation of the abdominal region with a hand-held blue lamp emitting light at a wavelength of 450 nm for a duration of 10 s. Quantification of phosphorescence luminescence and fluorescence was performed by ROI analysis of the interest region using Living Imaging software version 4.3.

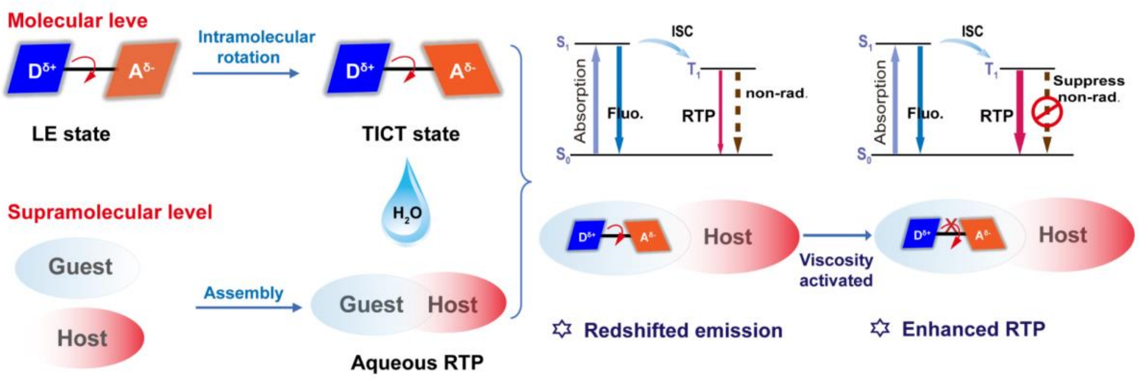

Scheme S1. Design principle of activatable RTP probe through TICT effect and host-guest strategy.

Synthesis and compound characterization

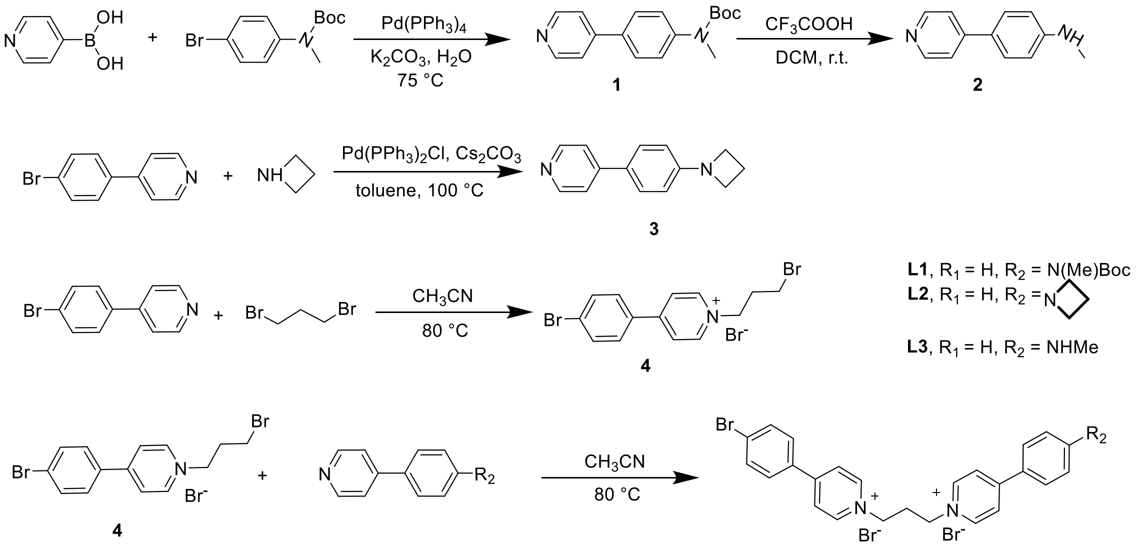

Scheme S2. Synthetic routes of guest molecule **Ln** (n=1-3).

Synthesis of compound 1. The tert-butyl (4-bromophenyl)(methyl)carbamate (1.1 g, 4 mmol), 4-pyridineboronic acid (0.55 g, 4.4 mmol), and K_2CO_3 (0.83 g, 6.0 mmol) were dissolved in anhydrous THF (40 mL) and degassed for 30 minutes.

Subsequently, $Pd(PPh_3)_4$ (0.19 g, 0.16 mmol) was rapidly added to the solution and degassing continued for an additional 15 minutes. The reaction mixture was heated at 110 ℃ under an argon atmosphere for a duration of 8 hours. The crude product was then poured into water (50 mL) and extracted with CH_2Cl_2 three times using portions of 20 mL each time. The organic layer was subsequently washed with saturated aqueous sodium bicarbonate solution three times using portions of 15 mL each time, followed by washing with saturated brine three times using portions of 20 mL each time. The organic layer was dried over anhydrous Na_2SO_4, filtered, and concentrated by rotary evaporation. Finally, the resulting residue underwent further purification through silica gel chromatography utilizing CH_2Cl_2: ethyl acetate as eluent in a ratio of 5:1 (v/v). Compound **5** was obtained as a white solid (0.58 g, 51%). ^1H NMR (400 MHz, $CDCl_3$) δ 8.65 (dd, *J* = 4.6, 1.6 Hz, 1H), 7.61 (d, *J* = 8.7 Hz, 1H), 7.49 (dd, *J* = 4.5, 1.7 Hz, 1H), 7.38 (d, *J* = 8.6 Hz, 1H), 3.31 (s, 2H), 1.49 (s, 5H). ^{13}C NMR (151 MHz, $CDCl_3$) δ 154.52, 150.22, 147.66, 144.70, 134.69, 127.11, 125.69, 121.41, 80.79, 37.13, 28.34.

Synthesis of compound 2. To a dry and clean 10 mL reaction flask, compound **1** (446.45 mg, 1.57 mmol) was added along with DCM (2 mL) and trifluoroacetic acid (1 mL). The mixture was continuously stirred at room temperature for 4 hours, followed by evaporation of the organic phase of DCM using a rotary evaporator to yield 254.55 mg of yellow solid product **2** (88% yield).

Synthesis of compound 3. The compound 4-(4-bromophenyl)pyridine (98.32 mg, 0.42 mmol), Cs_2CO_3 (411 mg, 1.26 mmol), $Pd(PPh_3)_2Cl_2$ (19 mg, 5% mmol), and azetidine (120 mg, 2.10 mmol) were dissolved in 5 mL of dry toluene under a nitrogen atmosphere. The resulting mixture was heated to 100 ℃ and stirred for a duration of 10 hours. Subsequently, the solvent was evaporated under reduced pressure and the remaining residue was further purified using column chromatography. (CH_2Cl_2: ethyl acetate, 5:1, V/V) to give 65 mg light yellow powder **3**, yield 74%. ^1H NMR (400 MHz, $CDCl_3$) δ 8.55 (dd, *J* = 4.6, 1.6 Hz, 2H), 7.54 (m, 2H), 7.45 (dd, *J* = 4.6, 1.7 Hz, 2H), 6.51 (m, 2H), 3.95 (t, *J* = 7.3 Hz, 4H), 2.41 (m, 2H). ^{13}C NMR (151 MHz, $CDCl_3$) δ 152.63, 149.82, 148.42, 127.57, 125.96, 120.47, 111.46, 52.21, 16.85.

Synthesis of compound 4. Compound **4** was synthesized following the procedure described in literature reference[1]. A solution of 4-(4-bromophenyl)pyridine (100 mg, 0.43 mmol) and 1,3-dibromopropane (0.35 g, 1.74 mmol) in CH_3CN (25 mL) was heated at 60 ℃ for a duration of 12 hours. After cooling to room temperature, the reaction mixture was dispersed in diethyl ether (250 mL). The resulting mixture was filtered and the solid product was washed with acetone. The final product was obtained as a white solid with a yield of 120 mg (66%).

Synthesis of compound L1. A mixture of Compound **1** (71 mg, 0.25 mmol) and Compound **4** (100 mg, 0.23 mmol) was dissolved in 10 mL CH_3CN and subjected to heating at 80 ℃ for a duration of 24 hours. The resulting residue was cooled to room temperature and subsequently evaporated to remove the solvent. The crude product was recrystallized using CH_3CN, yielding a white powder with an overall yield of 36% (60 mg). ^{1}H NMR (400 MHz, D_2O) δ 8.69 (dd, *J* = 14.8, 7.0 Hz, 4H), 8.09 (t, *J* = 7.2 Hz, 4H), 7.63 (d, *J* = 8.8 Hz, 1H), 7.56 (d, *J* = 2.5 Hz, 2H), 7.30 (d, *J* = 8.8 Hz, 2H), 4.79 (d, *J* = 6.2 Hz, 2H), 3.23 (s, 2H), 2.85 (m, 1H), 1.40 (s, 5H). ^{13}C NMR (151 MHz, CD_3OD) δ 156.00, 155.75, 154.40, 147.66, 144.85, 144.57, 132.81, 132.79, 129.88, 129.60, 128.31, 126.98, 125.74, 124.92, 124.42, 81.22, 57.25, 57.01, 36.00, 32.02, 27.13. HRMS (ESI) m/z for $C_{31}H_{34}Br_3N_3O_2$ calcd. $[M\text{-}2Br]^{2+}$ 279.5912, found: 279.5901.

Synthesis of compound L2. Similar to the synthesis of compound **L1**, compound **L2** was obtained as red powder with 36% yield (54 mg) with compound **3** (52.5 mg, 0.25 mmol) and compound **4** (100 mg, 0.23 mmol) as raw material. ^{1}H NMR (400 MHz, D_2O) δ 8.62 (d, *J* = 7.0 Hz, 2H), 8.30 (d, *J* = 7.1 Hz, 2H), 8.00 (d, *J* = 7.0 Hz, 2H), 7.75 (d, *J* = 7.2 Hz, 2H), 7.47 (dt, *J* = 11.9, 8.8 Hz, 6H), 6.31 (d, *J* = 8.9 Hz, 2H), 4.76 (d, *J* = 6.1 Hz, 2H), 4.62 (s, 2H), 3.93 (t, *J* = 7.5 Hz, 4H), 2.79 (s, 2H), 2.34 (m, 2H). ^{13}C NMR (151 MHz, $MeOD_4$) δ 155.94, 155.68, 154.46, 144.80, 143.22, 132.77, 132.73, 129.56, 129.39, 126.96, 124.88, 124.81, 121.16, 119.37, 110.74, 57.42, 56.11, 51.10, 31.79, 15.89. HRMS (ESI) m/z for $C_{28}H_{28}Br_3N_3$ calcd. $[M\text{-}2Br]^{2+}$ 242.5728, found: 242.5717.

Synthesis of compound L3. Similar to the synthesis of compound **L1**, compound **L3**

was obtained as orange powder with 34% yield (48 mg) with compound **2** (46 mg, 0.25 mmol) and compound **4** (100 mg, 0.23 mmol) as raw material. ^{1}H NMR (400 MHz, D_2O) δ 8.63 (d, *J* = 6.9 Hz, 2H), 8.29 (d, *J* = 7.0 Hz, 2H), 8.03 (d, *J* = 6.9 Hz, 2H), 7.76 (d, *J* = 7.1 Hz, 2H), 7.49 (dt, *J* = 8.7, 7.7 Hz, 4H), 6.52 (d, *J* = 9.0 Hz, 2H), 4.76 (s, 2H), 4.62 (s, 2H), 2.79 (s, 1H), 2.76 (s, 1H). ^{13}C NMR (151 MHz, $MeOD_4$) δ 155.83, 155.62, 154.49, 144.85, 143.16, 132.81, 132.79, 132.75, 132.74, 129.72, 129.60, 129.57, 129.56, 126.91, 124.90, 120.85, 118.92, 112.11, 57.42, 56.00, 31.86, 28.38. HRMS (ESI) m/z for $C_{28}H_{28}Br_3N_3$ calcd. $[M-2Br]^{2+}$ 229.5650, found: 229.5644.

Table S1. The viscosity of the water-glycerol mixture in different proportions at (25 ± 0.1) ℃.

glycerol (v%)	water (v%)	viscosity (cP)
0	100	0.82
10	90	1.03
20	80	1.24
30	70	1.58
40	60	2.58
50	50	4.34
60	40	7.79
70	30	17.36
80	20	43.09
90	10	113.00
95	5	326.27

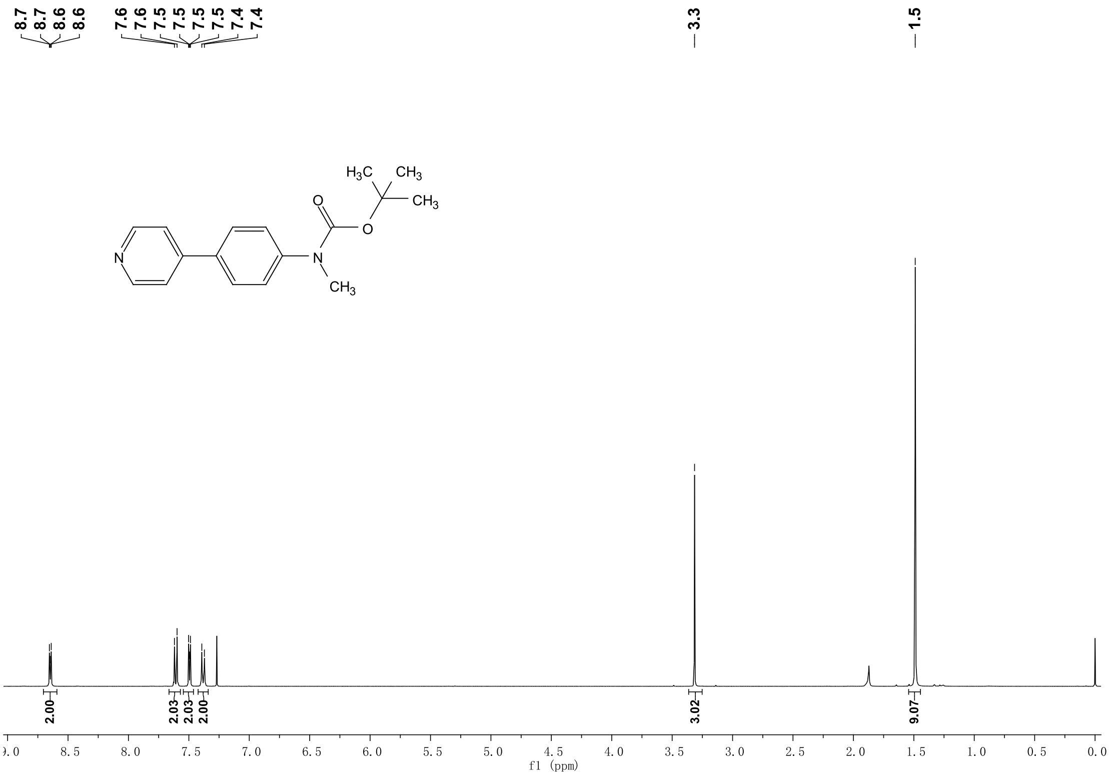

Figure S1. ^{1}H NMR spectrum of compound **1** in $CDCl_3$.

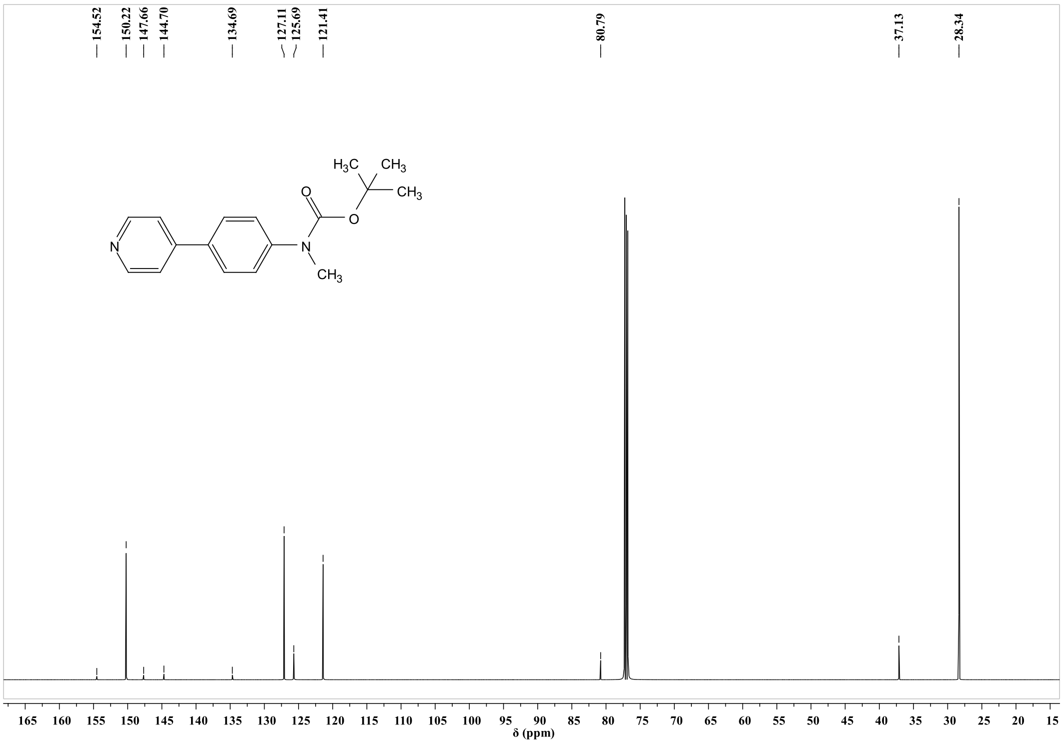

Figure S2. ^{13}C NMR spectrum of compound **1** in $CDCl_3$.

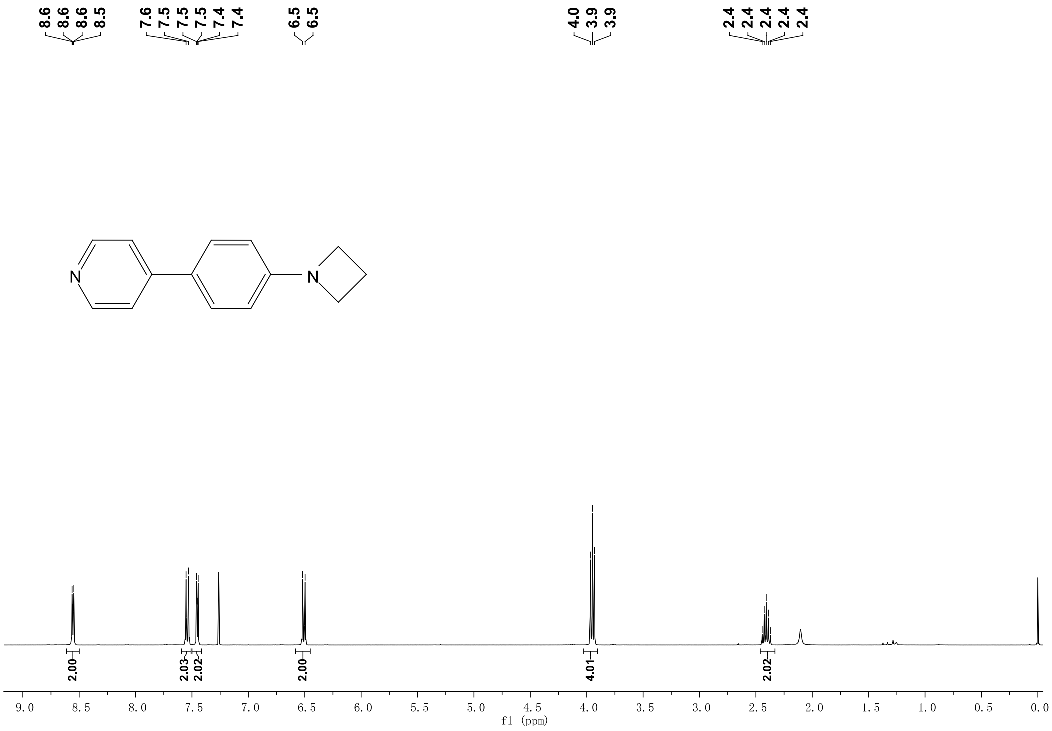

Figure S3. ^{1}H NMR spectrum of compound **2** in $CDCl_3$.

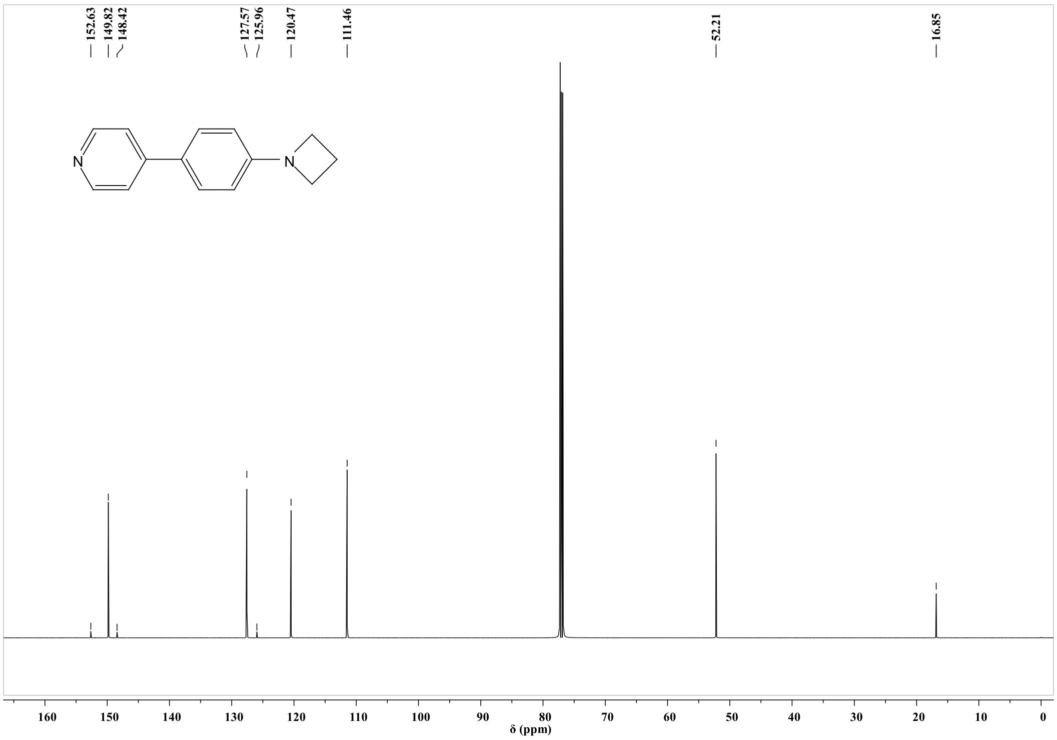

Figure S4. ^{13}C NMR spectrum of compound **2** in $CDCl_3$.

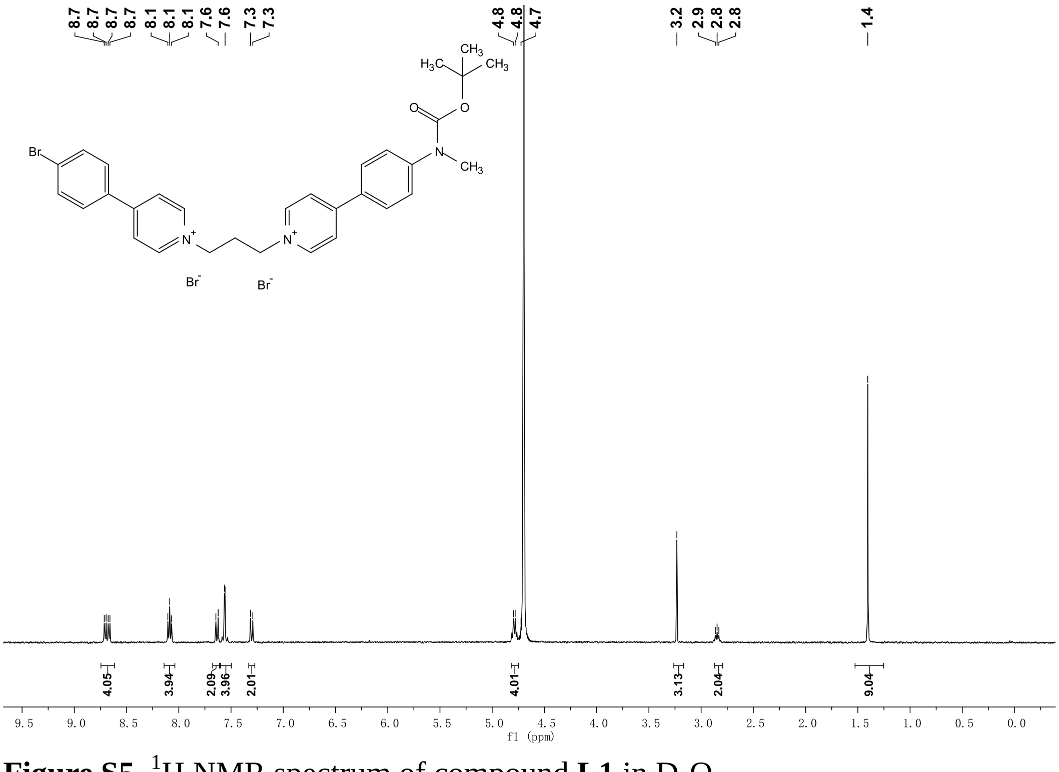

Figure S5. ^{1}H NMR spectrum of compound **L1** in D_2O.

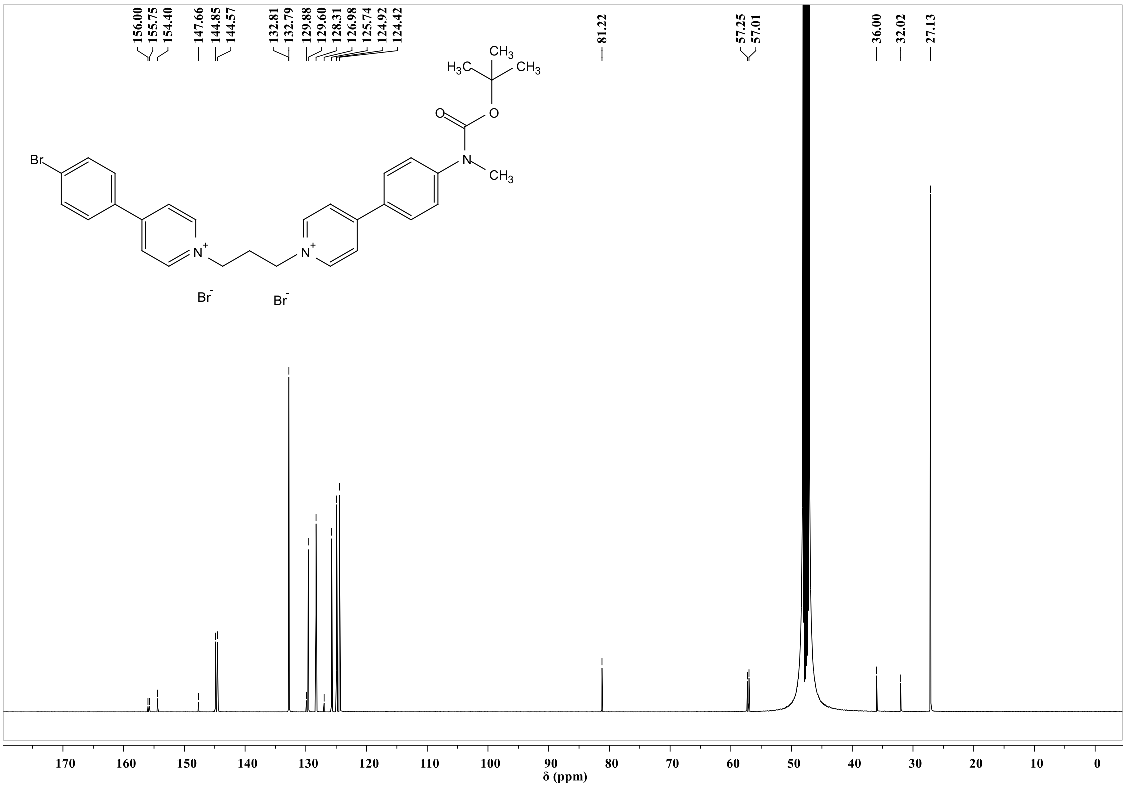

Figure S6. ^{13}C NMR spectrum of compound **L1** in CD_3OD.

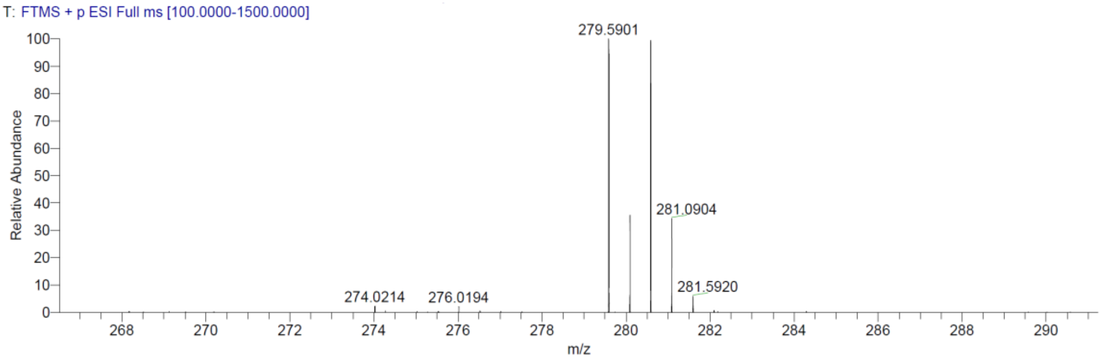

MX-WZ-302#422-478 RT: 2.14-2.42 AV: 57
T: FTMS + p ESI Full ms [100.0000-1500.0000]
m/z= 279.40-279.76

m/z	Intensity	Relative	Theo. Mass	Delta (ppm)	Composition
279.5901	1086499456.0	100.00	279.5912	-1.11	$C_{31}H_{34}O_2N_3Br$

Figure S7. HRMS spectrum of compound **L1**.

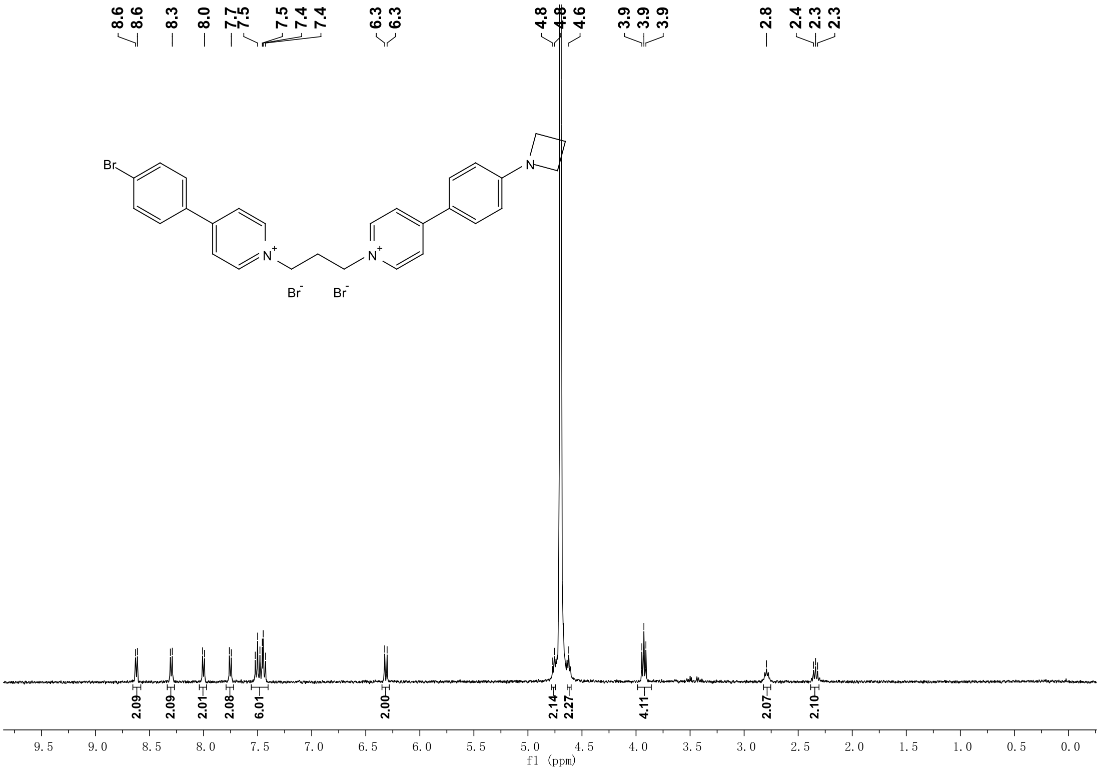

Figure S8. ^{1}H NMR spectrum of compound **L2** in D_2O.

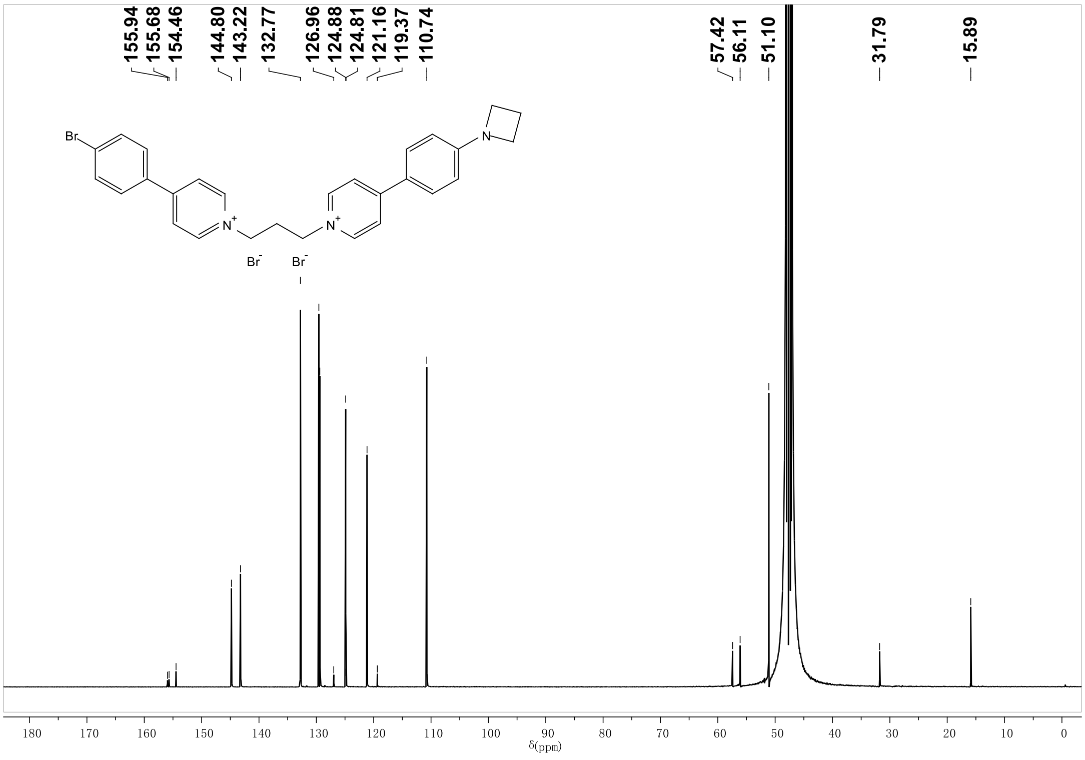

Figure S9. ^{13}C NMR spectrum of compound **L2** in CD_3OD.

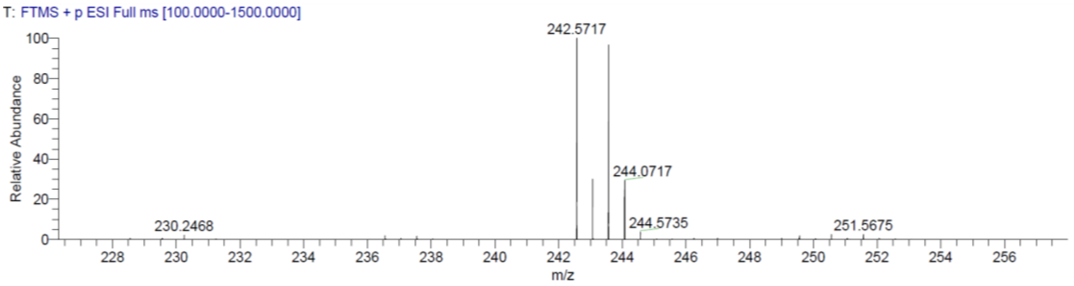

MX-LY-01#402-474 RT: 2.35-2.71 AV: 73
T: FTMS + p ESI Full ms [100.0000-1500.0000]
m/z= 242.03-242.76

m/z	Intensity	Relative	Theo. Mass	Delta (ppm)	Composition
242.5717	457197024.0	100.00	242.5728	-4.48	$C_{28}H_{28}N_3Br$

Figure S10. HRMS spectrum of compound **L2**.

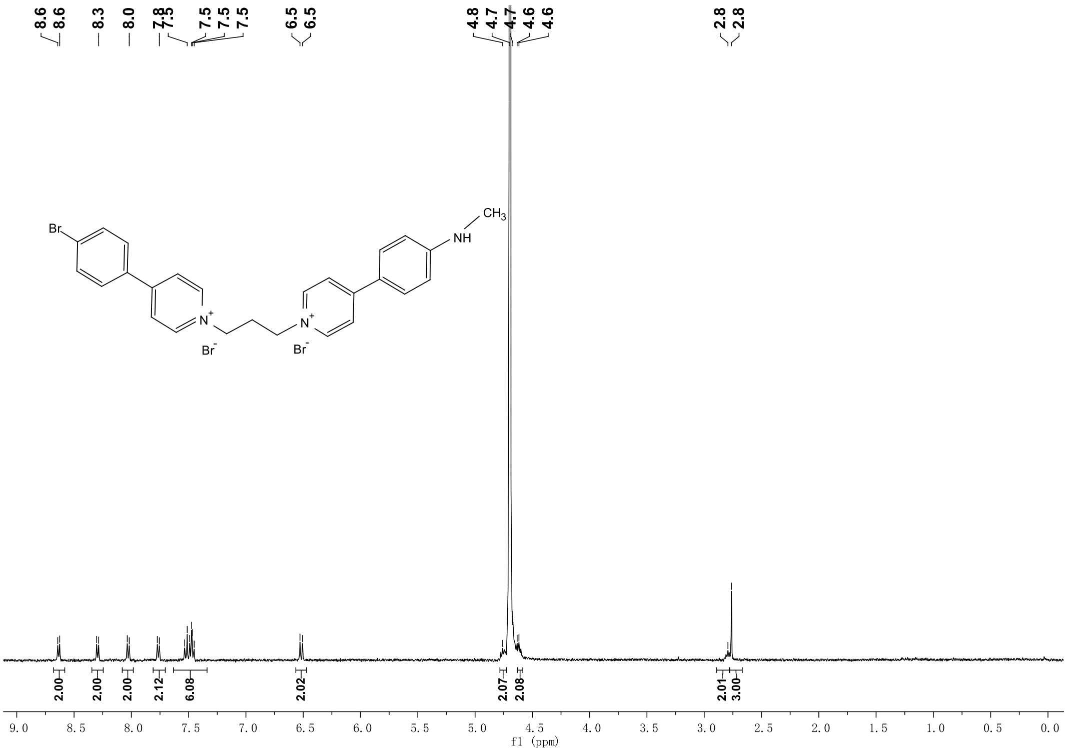

Figure S11. ^{1}H NMR spectrum of compound **L3** in D_2O.

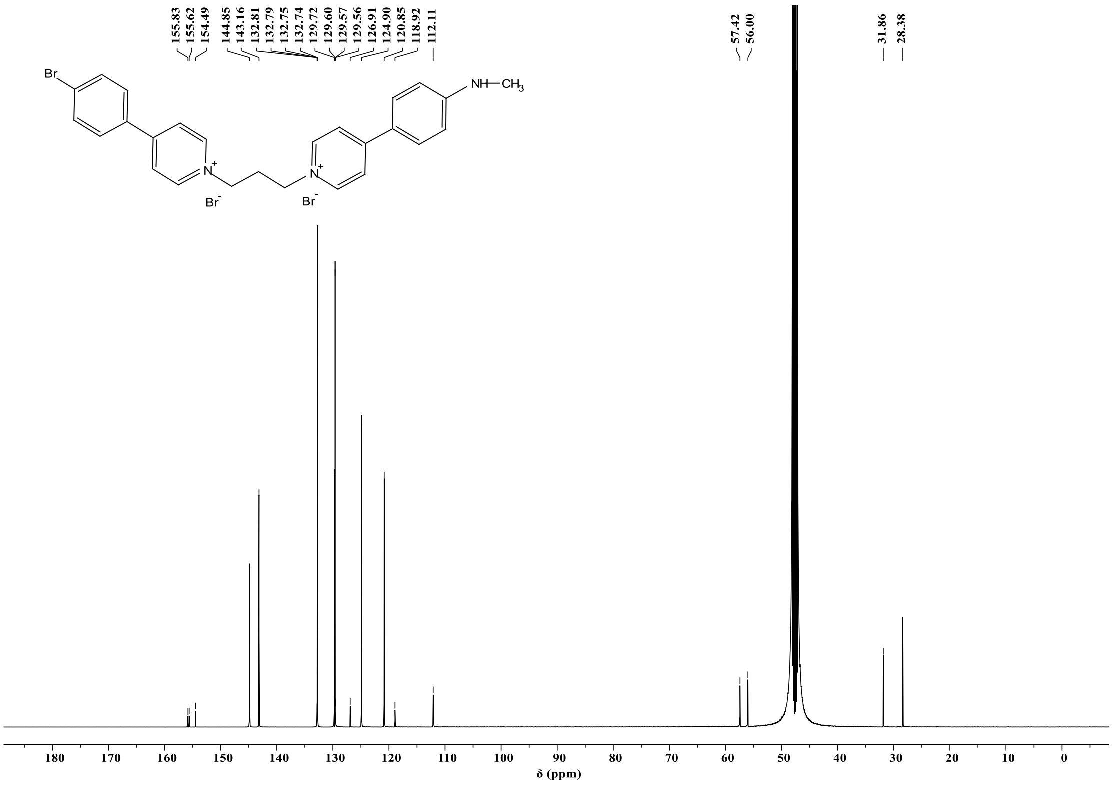

Figure S12. ^{13}C NMR spectrum of compound **L3** in CD_3OD.

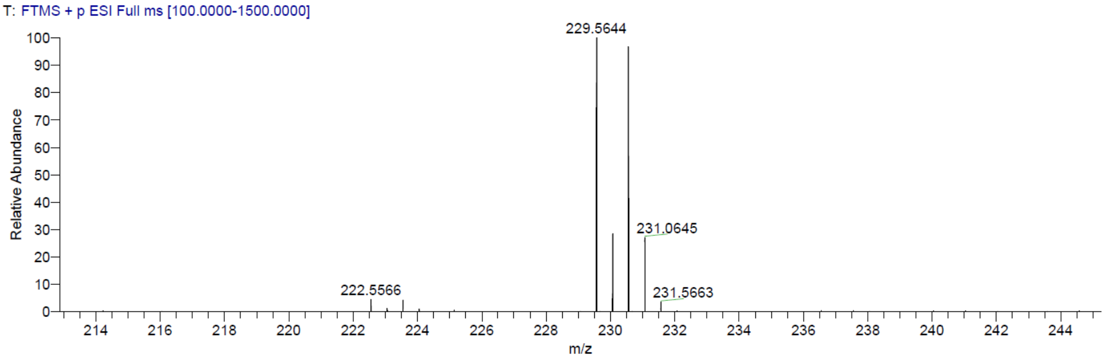

MX-WZ-303#436-455 RT: 2.20-2.30 AV: 20
T: FTMS + p ESI Full ms [100.0000-1500.0000]
m/z= 228.90-229.72

m/z	Intensity	Relative	Theo. Mass	Delta (ppm)	Composition
229.5644	1855528192.0	100.00	229.5650	-0.53	$C_{26}H_{26}N_3Br$

Figure S13. HRMS spectrum of compound **L3**.

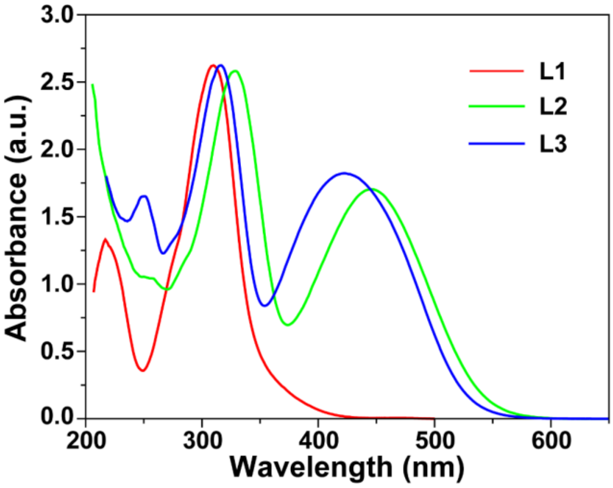

Figure S14. Absorption spectra of **L1**, **L2**, and **L3** in water (0.1 mM).

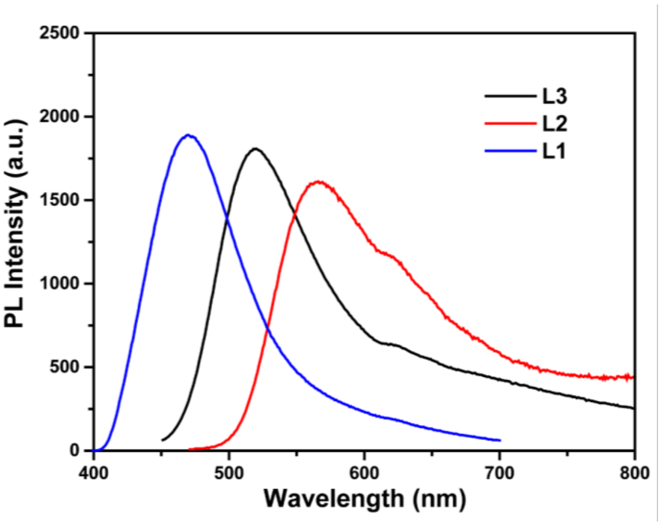

Figure S15. PL spectra of **L1** (excitation wavelength: 310 nm), **L2**(excitation wavelength: 420 nm), and **L3** (excitation wavelength: 425 nm) in water (0.1 mM).

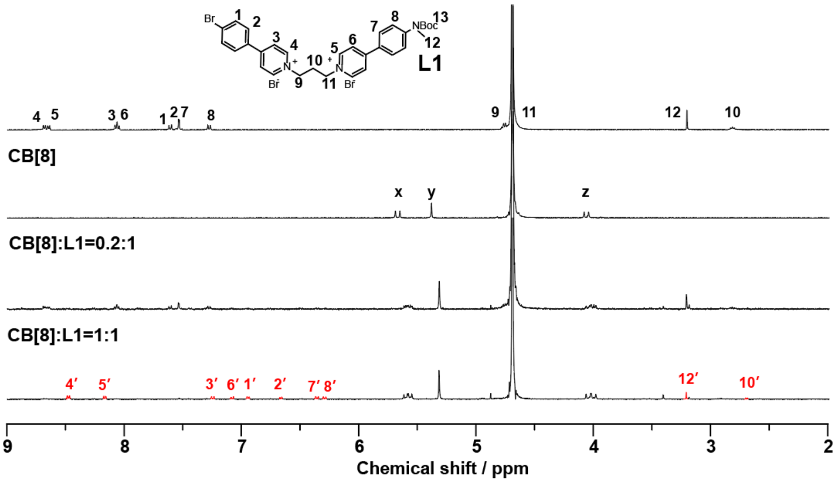

Figure S16. ^{1}H NMR spectral changes of **L1** after adding 0, 0.2 and 1.0 equivalent

CB[8] (400 MHz, D_2O).

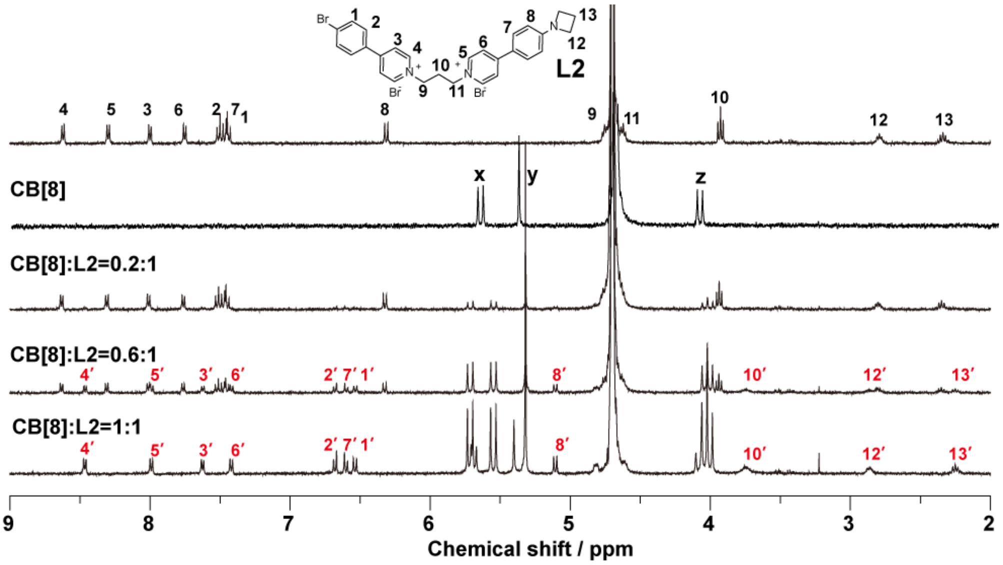

Figure S17. ^{1}HNMR spectral changes of **L2** after adding 0, 0.2 0.6, and 1.0 equivalent CB[8] (400 MHz, D_2O).

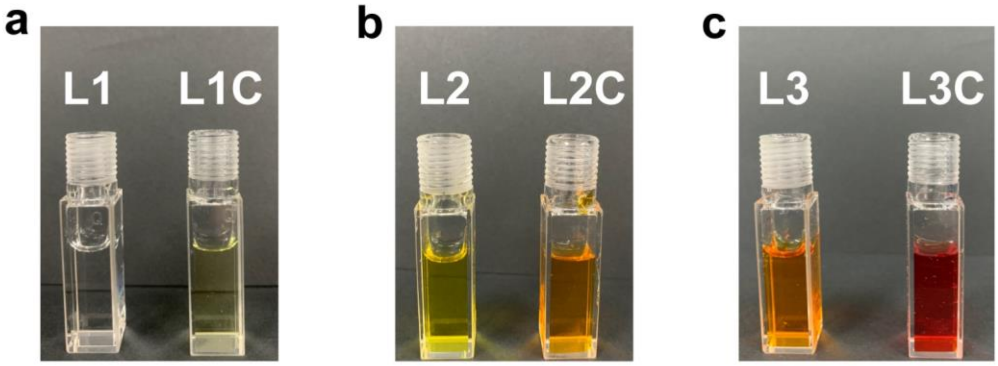

Figure S18. Color changes before and after assembly of **L1** (a), **L2** (b), and **L3** (c) with macrocyclic CB[8] in a 1:1 ratio in aqueous solution (0.1 mM).

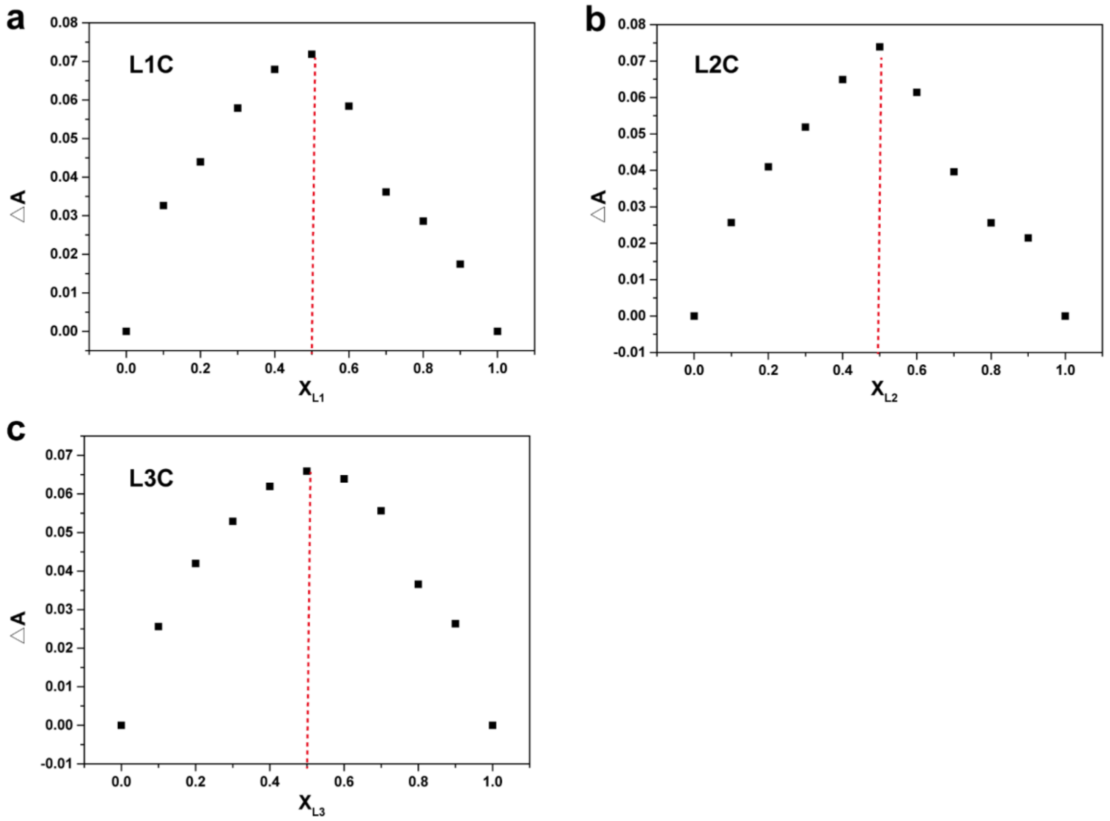

Figure S19. Job plot for **LnC** (n=3) and CB[8] in aqueous solution at 298 K by recording the absorbance at 310 nm. The total concentration is constant (**LnC** + [CB[8]] = 2.0 × 10^{-5} M).

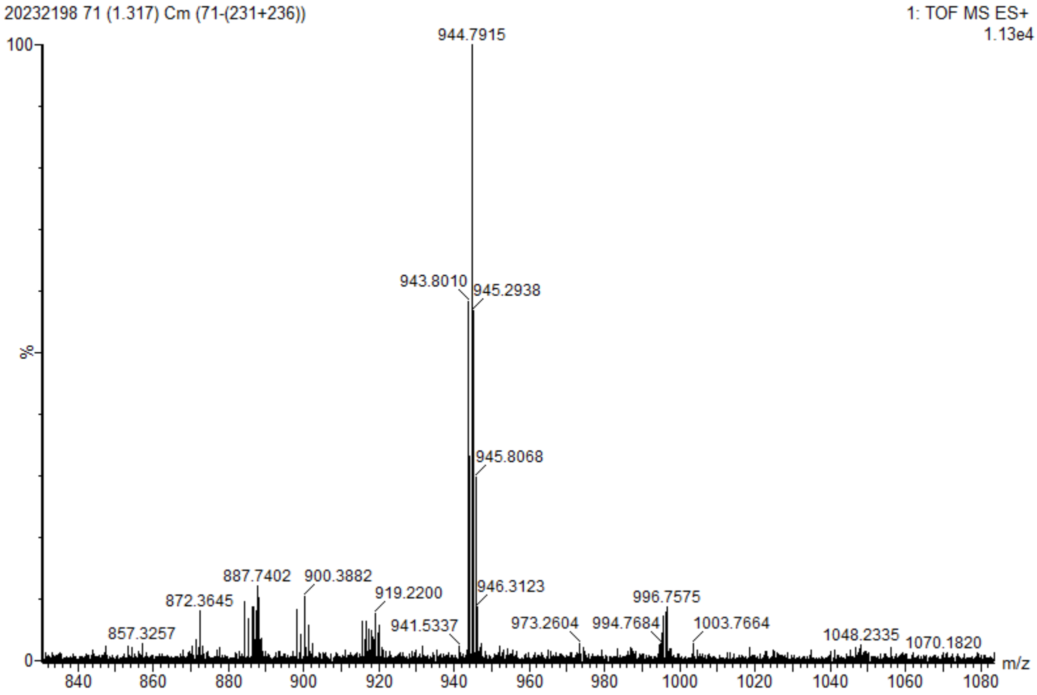

Figure S20. MOLDI-TOF-MS spectrum of compound **L1C**.

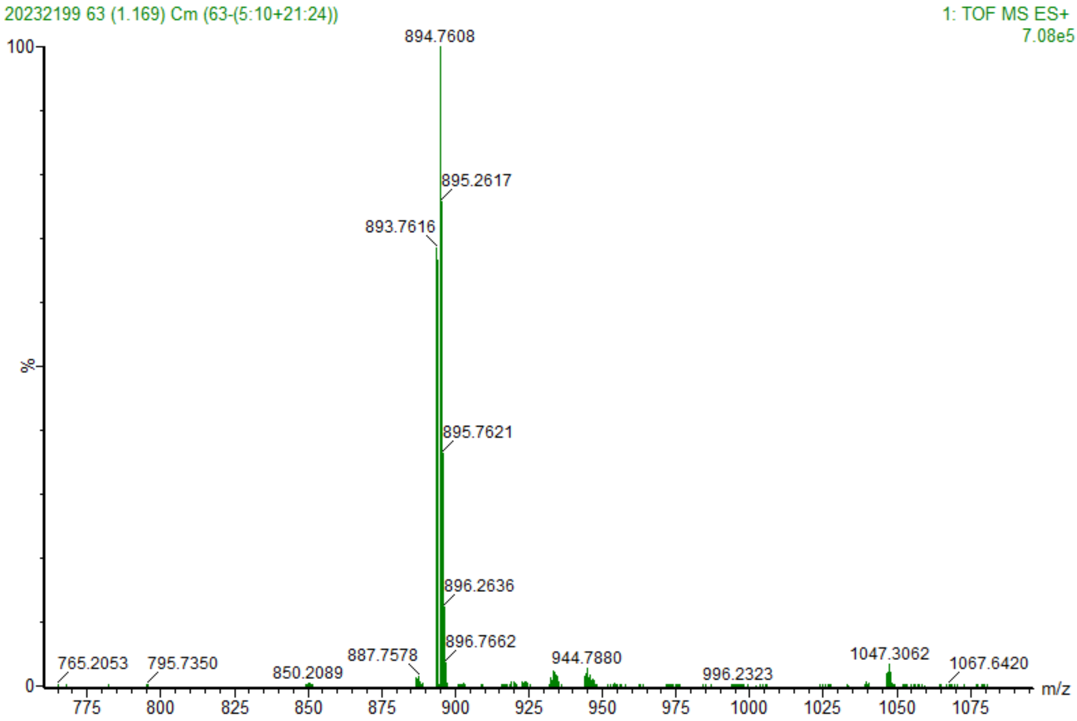

Figure S21. MOLDI-TOF-MS spectrum of compound **L2C**.

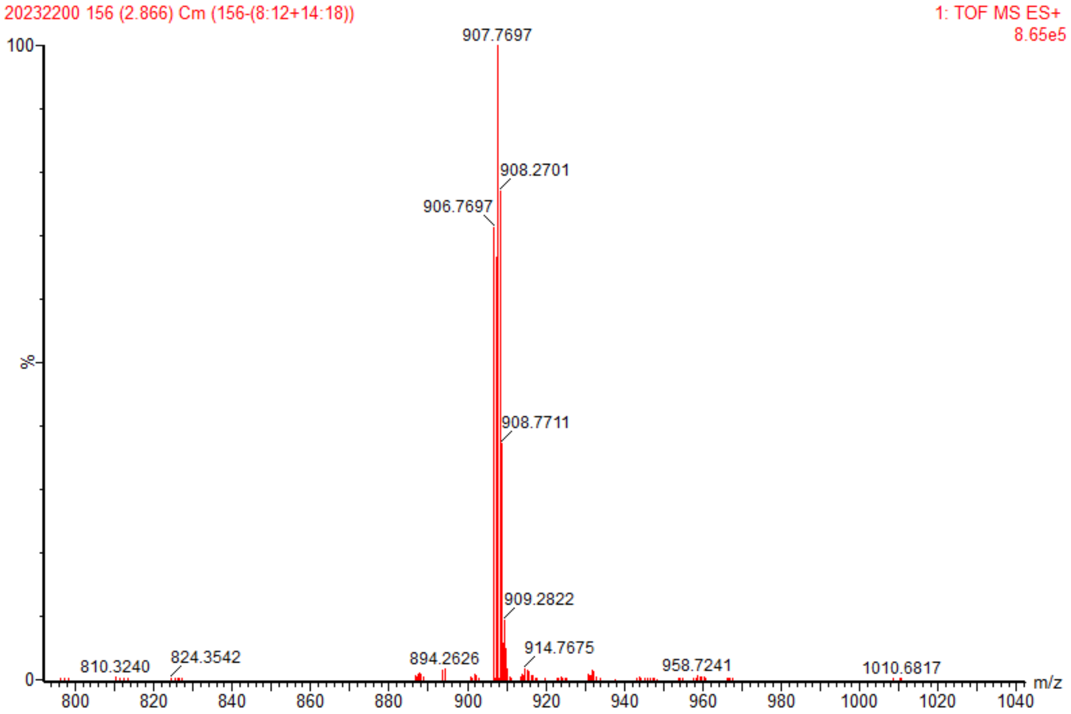

Figure S22. MOLDI-TOF-MS spectrum of compound **L3C**.

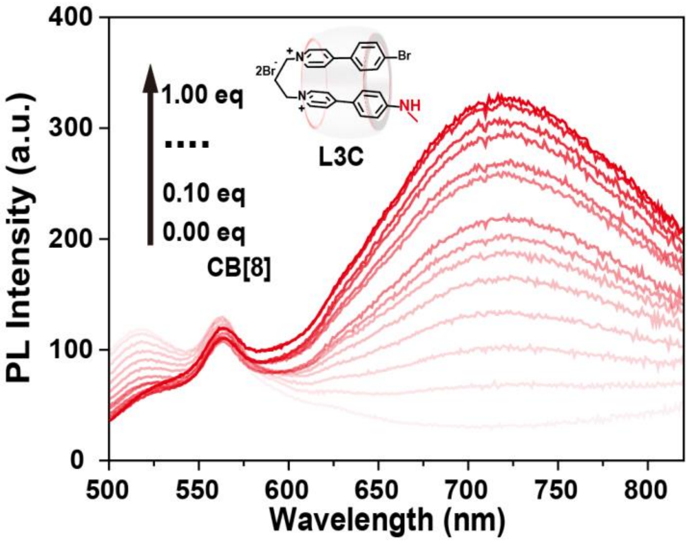

Figure S23. PL spectra of aqueous solutions of **L3** (10×10^{-6} m) and CB[8] (excited by 428 nm) at concentrations ranging from 0 to 12×10^{-6} m under ambient conditions.

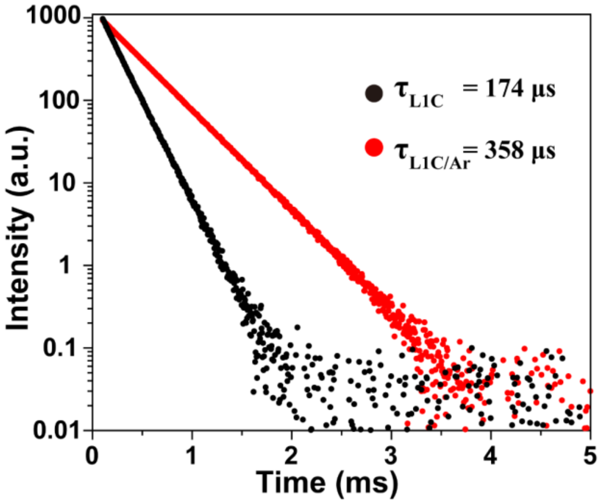

Figure S24. The time delay of **L1C** under argon in water at 298 K (**L1C**=1×10^{-4} M).

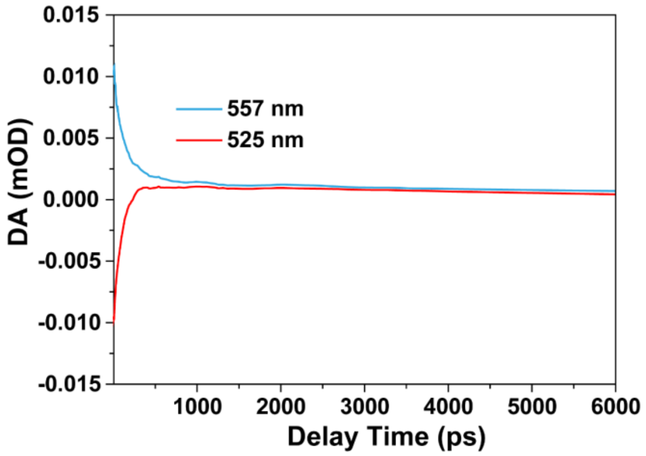

Figure S25. Dynamics of **L2C** at 557 and 525 nm detection wavelengths.

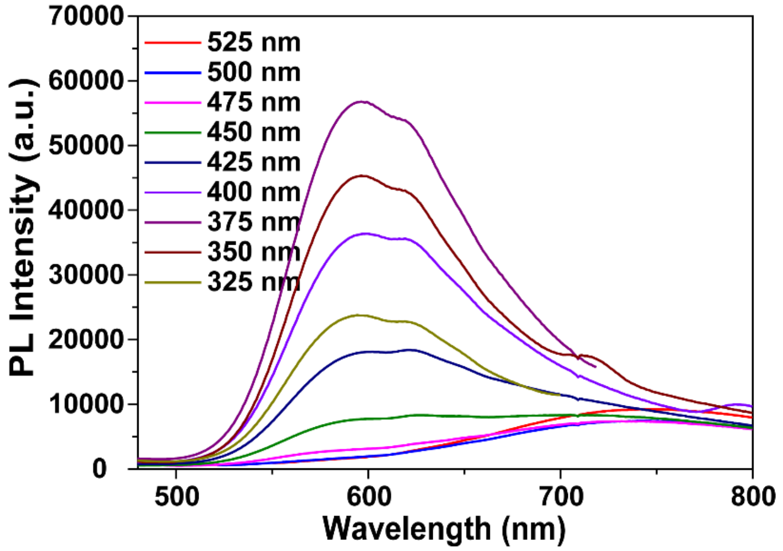

Figure S26. PL spectra of **L2C** (1×10^{-4} M) in aqueous solution under ambient conditions excited with different wavelengths (from 325 to 525 nm).

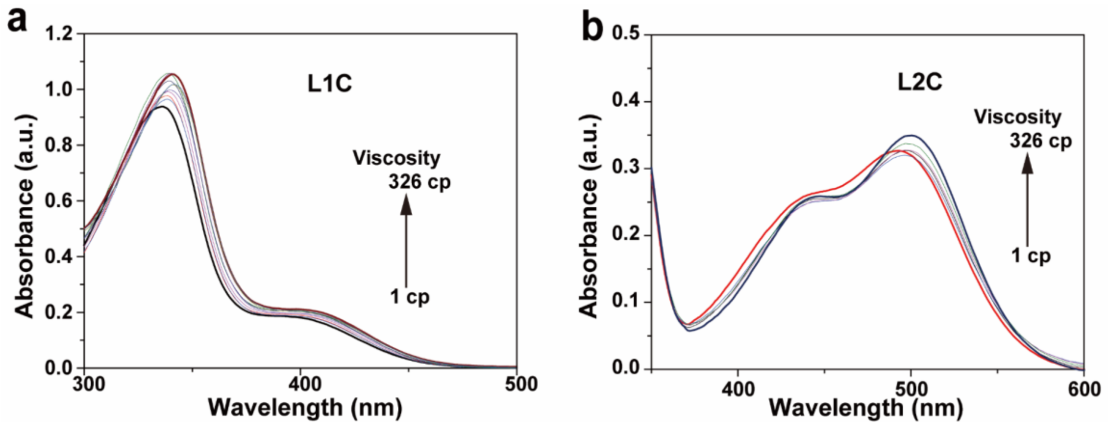

Figure S27. Absorption spectra of **L1C** and **L2C** (5×10^{-5} M) in water/glycerol mixtures with different viscosities.

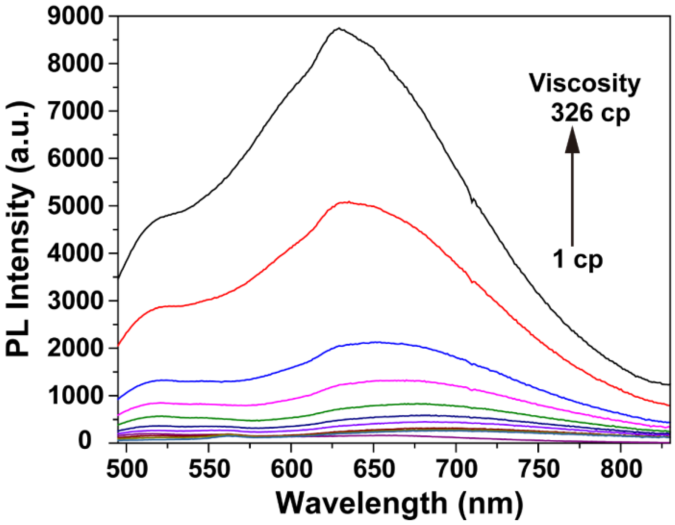

Figure S28. PL spectra of **L3C** (5×10^{-5} M) in water/glycerol mixtures with different viscosities in the emission window of 500−850 nm (excitation wavelength: 470 nm).

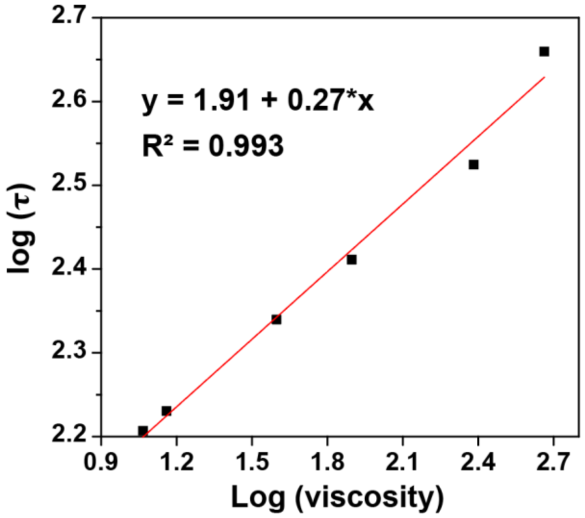

Figure S29. Linear relationship between log τ and log (viscosity). λ_{ex} = 400 nm, λ_{em} = 585 nm.

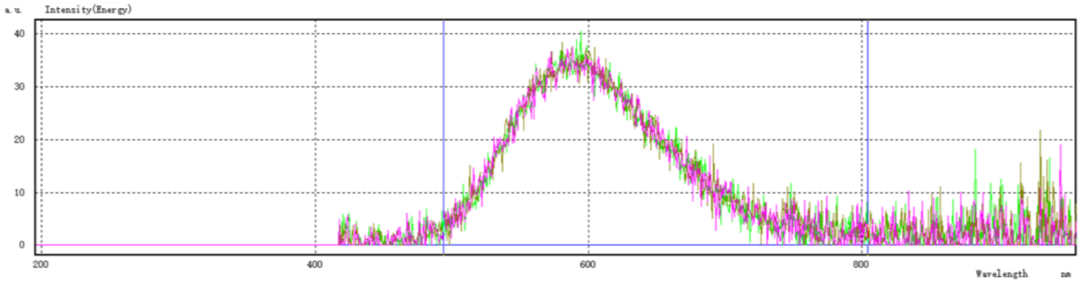

N ...	Cursor1-2: 395.54 - 416.67(...	Cursor3-4: 494.13 - 804.52(...	QuantumYield	QY(Corrected)	Factor a	Abs	Peak Wavele...	Peak Co...	Peak FW...	Peak Wavele...	Peak Co...	Peak FW...	RMS
1	115141875	---	---	---	---	---	406.11	47260.87	5.73	496.37	10.63	5.92	---
2	52937012	2825614	0.045	---	---	0.540	406.11	21648.60	5.75	594.21	39.87	101.89	---
3	52971644	2906228	0.047	---	---	0.540	406.11	21702.29	5.75	580.81	39.12	114.67	---
4	52943095	2838869	0.046	---	---	0.540	406.11	21701.35	5.74	584.53	38.43	124.29	---

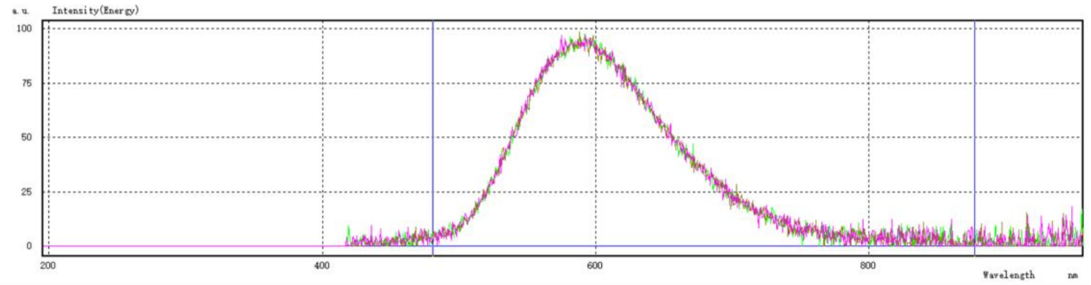

N ...	Cursor1-2: 395.54 - 416.67(...	Cursor3-4: 480.62 - 877.48(...	QuantumYield	QY(Corrected)	Factor a	Abs	Peak Wavele...	Peak Co...	Peak FW...	Peak Wavele...	Peak Co...	Peak FW...	RMS
1	111769137	---	---	---	---	---	406.11	45722.48	5.75	490.38	13.66	2.90	---
5	42490033	7622497	0.110	---	---	0.620	406.11	17315.70	5.77	591.98	98.17	120.12	---
5	42442085	7557863	0.109	---	---	0.620	406.11	17276.53	5.77	588.26	99.73	118.99	---
5	42394212	7648776	0.110	---	---	0.621	406.11	17274.64	5.77	574.85	98.18	118.66	---

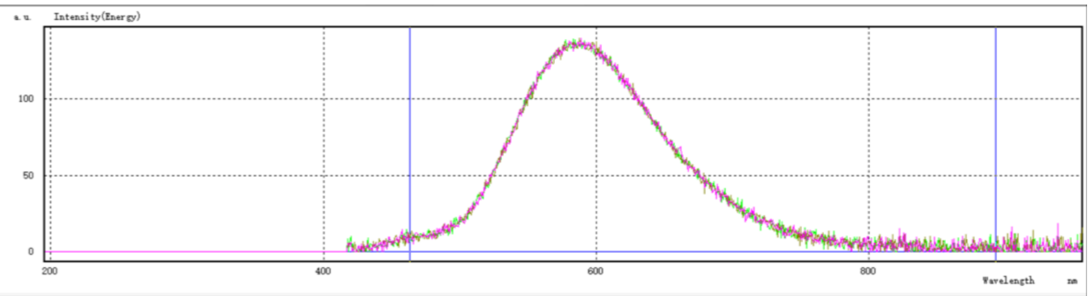

N ...	Cursor1-2: 395.54 - 416.67(...	Cursor3-4: 463.35 - 892.75(...	QuantumYield	QY(Corrected)	Factor a	Abs	Peak Wavele...	Peak Co...	Peak FW...	Peak Wavele...	Peak Co...	Peak FW...	RMS
1	106516782	---	---	---	---	---	406.11	43762.33	5.72	469.36	19.32	179.65	---
2	46364834	11046363	0.184	---	---	0.565	406.11	18797.97	5.81	583.05	141.63	120.68	---
3	46289879	11090193	0.184	---	---	0.565	406.11	18780.49	5.80	587.51	140.12	122.11	---
4	46199268	11068982	0.184	---	---	0.566	406.11	18778.07	5.79	589.00	140.35	122.37	---

Figure S30. The phosphorescence quantum yield of **L1C** in water and water/glycerol mixtures.

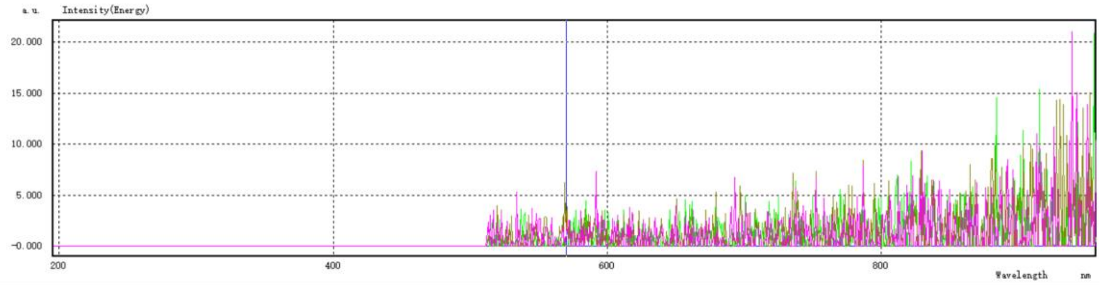

N ...	Cursor1-2: 490.38 - 511.36(...	Cursor3-4: 570.38 - 957.25(...	QuantumYield	QY(Corrected)	Factor a	Abs	Peak Wavele...	Peak Co...	Peak FW...	Peak Wavele...	Peak Co...	Peak FW...	RMS
1	120691399	---	---	---	---	---	500.87	42484.38	5.47	952.19	14.91	0.55	---
2	33968720	181258	0.002	---	---	0.719	500.87	11929.43	5.49	955.80	21.64	0.92	---
3	33979790	229719	0.003	---	---	0.718	500.87	11919.41	5.50	947.85	14.52	0.72	---
4	33942632	235358	0.003	---	---	0.719	500.87	11906.65	5.49	939.89	17.98	1.09	---

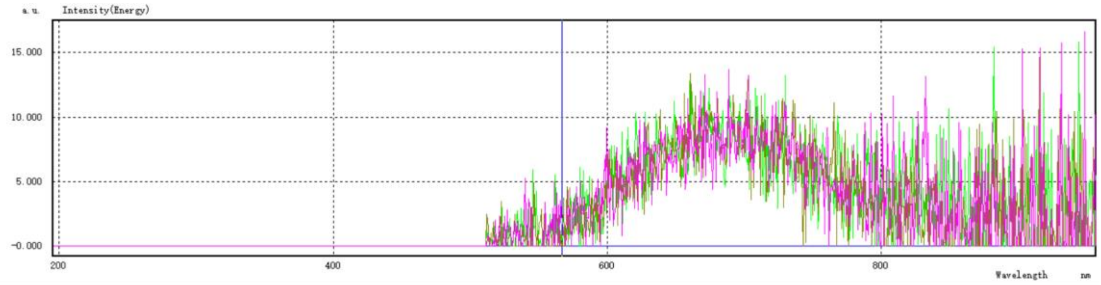

N ...	Cursor1-2: 490.38 - 511.36(...	Cursor3-4: 566.65 - 957.25(...	QuantumYield	QY(Corrected)	Factor a	Abs	Peak Wavele...	Peak Co...	Peak FW...	Peak Wavele...	Peak Co...	Peak FW...	RMS
1	117186342	---	---	---	---	---	500.87	41225.38	5.48	944.23	16.16	0.58	---
3	13583193	1319283	0.013	---	---	0.884	500.87	4708.88	5.55	944.95	15.75	0.96	---
4	13604973	1168605	0.011	---	---	0.884	501.62	4695.53	5.57	915.98	13.89	0.91	---
5	13731004	1193709	0.012	---	---	0.883	500.87	4740.04	5.57	949.29	17.34	0.54	---

Figure S31. The PL quantum yield of **L2C** in water and water/glycerol mixtures.

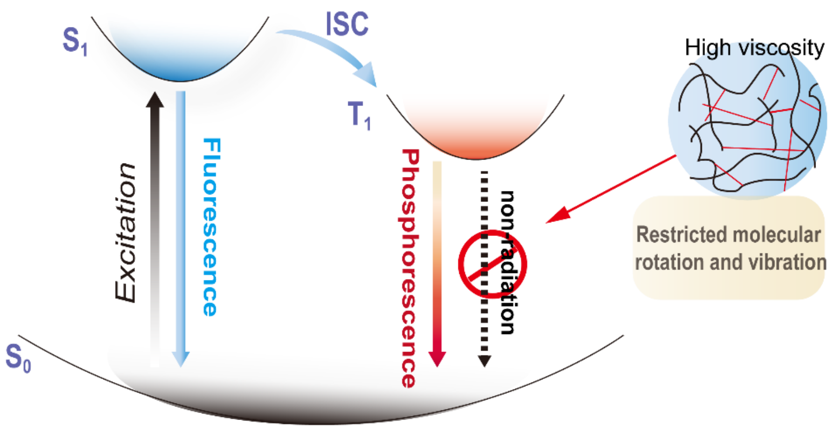

Figure S32. Simplified Jablonski diagrams illustrating fluorescence and phosphorescence emissions, along with the impact of molecular motion restriction induced by high viscosity.

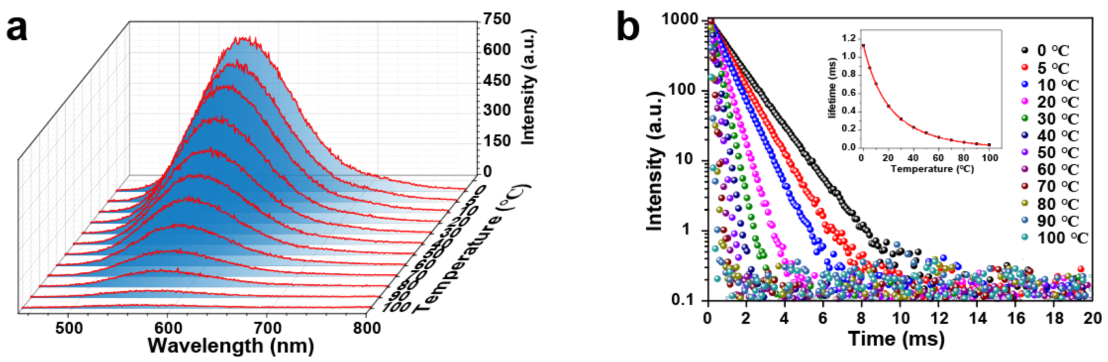

Figure S33. (a) Phosphorescence spectra of **L1C** (5 μM) in water/glycerol mixtures at different temperatures. (b) Phosphorescence lifetime spectra of **L1C** (10 μM) in the water/glycerol solutions, temperature ranging from 0 °C to 100 °C.

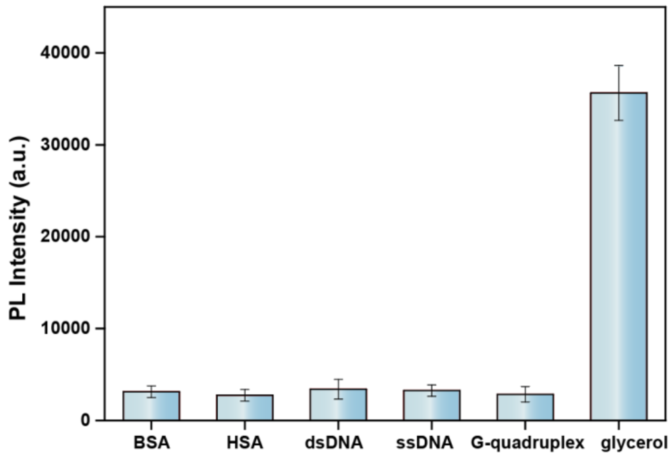

Figure S34. Phosphorescence response of **L1C** (50 μM) to various biomolecules (2

μM BSA, 2 μM HAS, 40 μg/mL ssDNA, 50 μg/mL dsDNA, 50 μg/mL and 10 μM G-quadruplex).

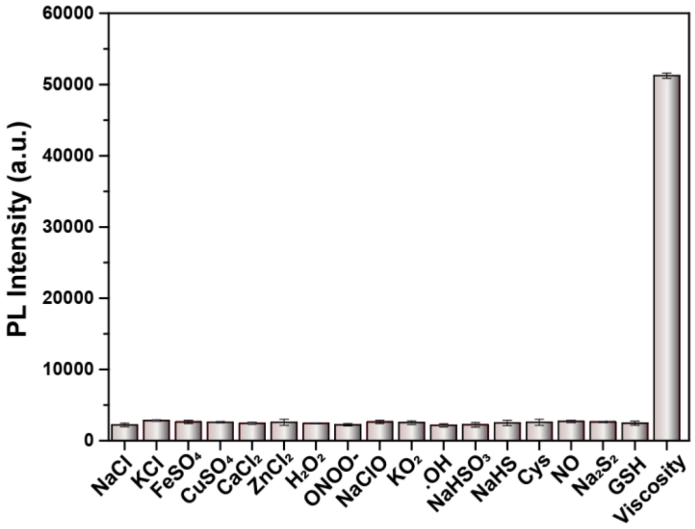

Figure S35. Phosphorescence response of **L2C** (50 μM) to various metal ions, ROS, and RNS.

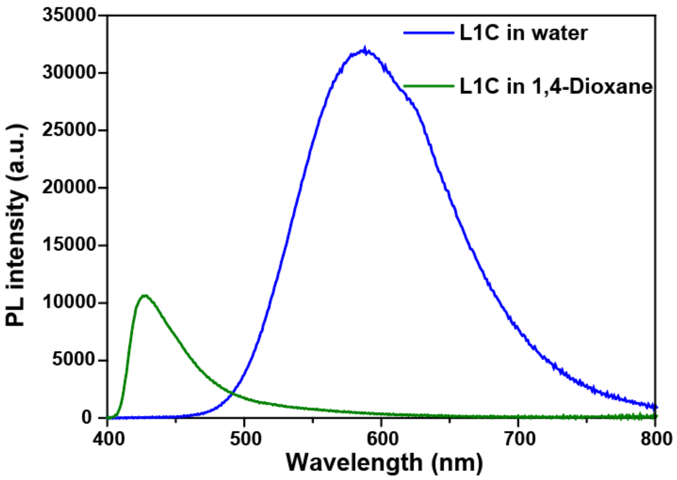

Figure S36. PL spectra of **L1C** (50 μM) at water and 1,4-Dioxane.

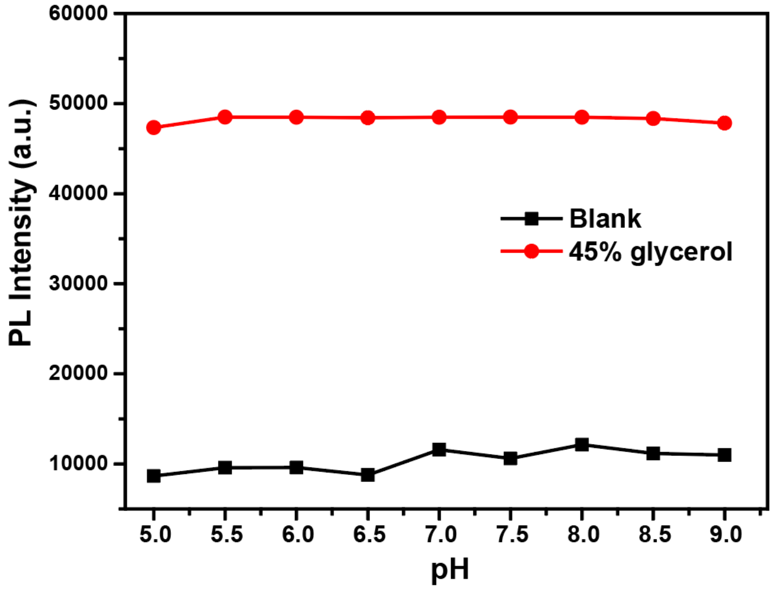

Figure S37. Fluorescence spectra of **L1C** (5×10^{-5} M) in PBS buffer (10 mM) and after the addition of glycerol (45%) at various pH. λ_{ex} = 400 nm.

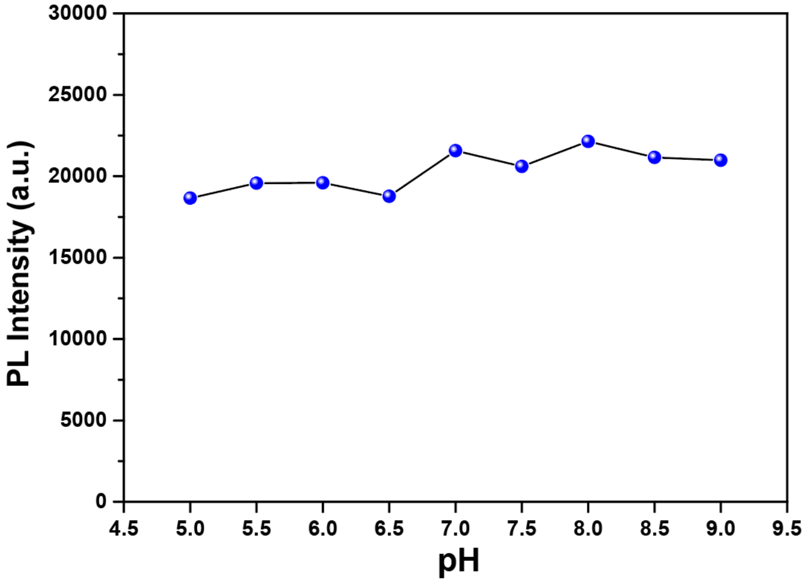

Figure S38. Fluorescence spectra of **L2C** (5×10^{-5} M) in PBS buffer (10 mM) at various pH. λ_{ex} = 500 nm.

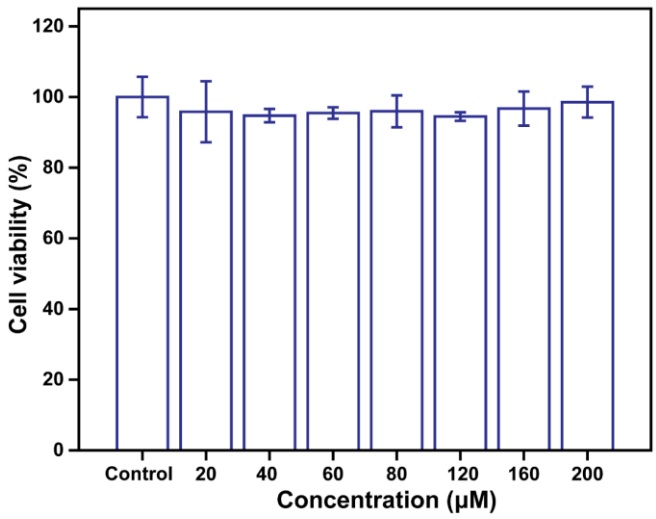

Figure S39. Cell toxicity of **L1C** towards HeLa cells with an incubation time of 24 h. Error bar represents s.d.

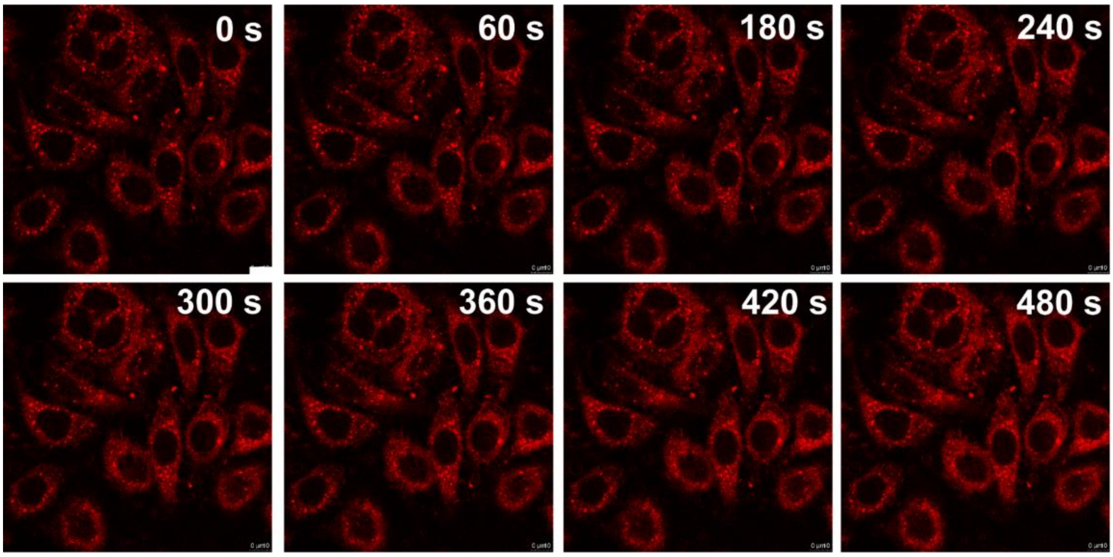

Figure S40. Intracellular photobleaching of RTP probe **L1C** a 405 nm laser (intensity 30 %) in HeLa cells. Phosphorescence images of HeLa cells stained with 100 μM **L1C** were captured after every 60 s irradiation.

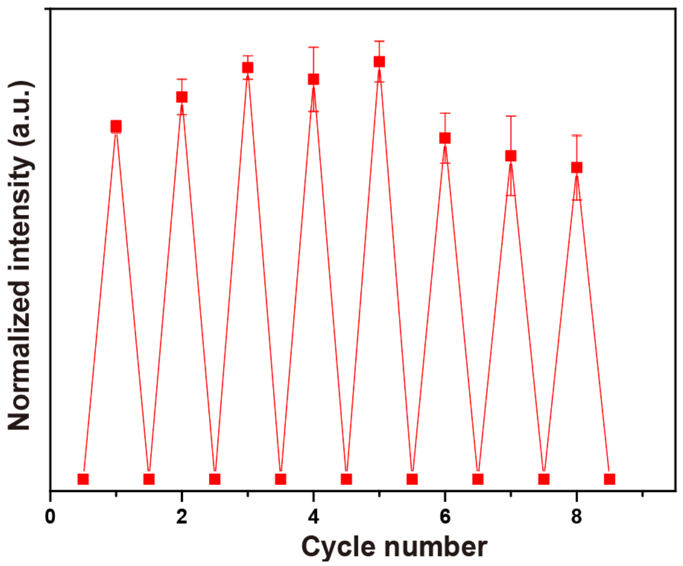

Figure S41. The normalized phosphorescence intensities of **L1C** as a function of cycle number of 450 nm light irradiation (n = 3).

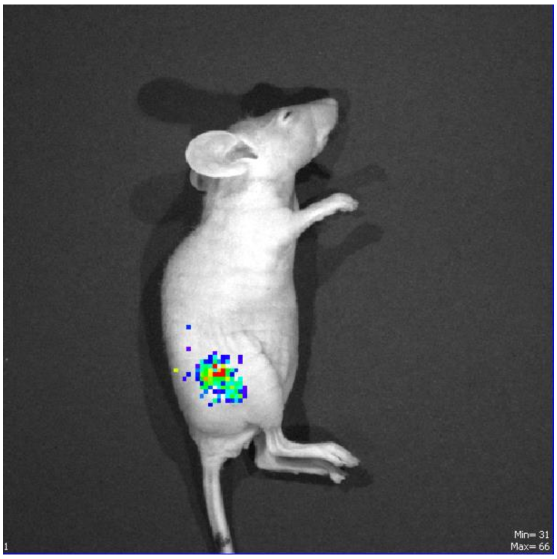

Figure S42. Phosphorescence after the subcutaneous injection of probe **L1C** (200 μM) to nude mouse.

Reference

1. Ma, X. K.; Zhang, W.; Liu, Z.; Zhang, H.; Zhang, B.; Liu, Y. Supramolecular Pins with Ultralong Efficient Phosphorescence. *Adv Mater.* **2021**, *33*, e2007476.
